# Functional Benefit and Safety of Adjunctive Intra-Arterial Thrombolysis After Successful Endovascular Thrombectomy: An Updated and Cumulative Meta-Analysis of Randomized Trials

**DOI:** 10.3390/jcm15207777

**Published:** 2026-10-09

**Authors:** Burcu Yagmur, Elif Ijlal Cekirdekci, Lutfi Cagatay Onar, Ibrahim Yilmaz

**Affiliations:** 1 Department of Cardiology, Faculty of Medicine, University of Kyrenia, Kyrenia 99320, Cyprus; 2 Department of Cardiovascular Surgery, Dr. Ismail Fehmi Cumalioglu City Hospital, Ministry of Health, Republic of Turkey, Tekirdag 59020, Turkey; 3 Unit of Pharmacovigilance, Dr. Ismail Fehmi Cumalioglu City Hospital, Ministry of Health, Republic of Turkey, Tekirdag 59020, Turkey

**Keywords:** intra-arterial thrombolysis, endovascular thrombectomy, acute ischemic stroke, large-vessel occlusion, fibrinolytic agents, alteplase, tenecteplase, urokinase, cumulative meta-analysis, fragility analysis

## Abstract

**Background/Objectives:** Adjunctive intra-arterial thrombolysis (IAT) after successful endovascular thrombectomy has been associated with a higher probability of excellent functional recovery in previous randomized-trial meta-analyses. Since those syntheses, the evidence base has expanded to include IAT-TOP, restricted to basilar artery occlusion, and CHOICE-2, in which 90-day mortality was 12.1% with adjunctive alteplase and 6.4% with thrombectomy alone. We therefore examined how the efficacy and mortality profiles changed as randomized evidence accumulated. **Methods:** This PRISMA 2020-compliant systematic review and meta-analysis was prospectively registered in PROSPERO (CRD420251006577). Eligible studies were randomized controlled trials (RCTs) comparing adjunctive intra-arterial fibrinolytic therapy administered after trial-defined successful or near-successful post-thrombectomy reperfusion with no adjunctive intra-arterial fibrinolysis. PubMed/MEDLINE, Embase, and CENTRAL were searched through 15 August 2026, with ClinicalTrials.gov searched separately. Nine RCTs comprising 2828 randomized participants were included at the systematic-review level. Event counts were re-extracted from primary trial publications and pooled as risk ratios (RRs) using random-effects models with restricted maximum likelihood estimation and Hartung–Knapp confidence intervals (CIs). Cumulative meta-analysis tracked how the efficacy and mortality estimates changed as evidence accumulated, while fragility analyses assessed the stability of the corresponding statistical inferences. This research received no external funding. **Results:** Adjunctive IAT increased excellent functional recovery (modified Rankin Scale [mRS] 0–1; 8 RCTs; RR 1.266, 95% CI 1.135–1.412; I^2^ = 0.0%), with a 95% prediction interval of 1.047–1.530 and a meta-analytic Fragility Index of 15. Ninety-day all-cause mortality did not differ significantly between groups (8 RCTs; RR 1.036, 95% CI 0.817–1.314); immediately before CHOICE-2 entered the cumulative synthesis, the corresponding estimate was RR 0.984 (95% CI 0.801–1.209). In the bidirectional reverse-fragility analysis, the earliest significance transition identified by the search occurred after 24 one-patient event-status modifications and was in the direction of increased mortality with adjunctive IAT (RR 1.174, 95% CI 1.002–1.377; *p* = 0.048). No transition toward reduced mortality was identified through the same modification depth. No trial was judged to be at high risk of bias using RoB 2. GRADE certainty was moderate for excellent functional recovery, 90-day mortality, and functional independence and low for hemorrhagic outcomes. **Conclusions:** The efficacy and mortality components of the benefit–risk profile of adjunctive IAT have not evolved in parallel. The improvement in excellent functional recovery persisted as randomized evidence accumulated and remained comparatively stable to perturbation of the observed event data, whereas mortality remains unresolved. The cumulative and reverse-fragility findings do not establish a treatment-related mortality excess; however, the absence of statistical significance should not be interpreted as affirmative evidence of mortality neutrality. The current randomized evidence therefore supports a reproducible functional benefit while preserving clinically relevant uncertainty regarding survival.

## 1. Introduction

Endovascular thrombectomy (EVT) has become the standard of care for acute ischemic stroke due to large-vessel occlusion (LVO), and its benefit relative to medical management alone is supported by individual-patient-data pooling of the pivotal randomized trials [1]. Yet the pharmacology of stroke recovery does not end at the point of angiographic success. A substantial proportion of patients who achieve successful or near-successful endovascular reperfusion—defined in the cited evidence using an expanded Treatment in Cerebral Ischemia (eTICI) threshold of grade 2b or higher—nonetheless fail to achieve functional independence at 90 days. This disjunction between macrovascular patency and clinical outcome has been attributed to persistent impairment of tissue-level perfusion despite a patent proximal vessel. In a post hoc pooled analysis of two perfusion-imaging trials, residual hypoperfusion negated essentially the entire clinical benefit otherwise associated with successful recanalization [2].

Mechanistically, this residual hypoperfusion is not a single pharmacologic target. A recent synthesis distinguishes two biologically distinct substrates: distal embolic territorial hypoperfusion, in which macroembolic fragments dislodged during mechanical thrombectomy occlude vessels beyond the reach of the retrieval device and remain, in principle, susceptible to pharmacologic thrombolysis; and true ischemic-core no-reflow, a capillary-level phenomenon driven by pericyte-mediated microvascular constriction, endothelial swelling, and thromboinflammatory occlusion that is unlikely to respond to any fibrinolytic agent, however delivered [3]. This distinction is pharmacologically important because it defines the plausible ceiling of benefit from adjunctive thrombolysis. It also provides a framework for interpreting the magnitude of effect observed in the present synthesis.

Adjunctive intra-arterial thrombolysis (IAT) was conceived as a pharmacokinetically rational intervention against the first of these two substrates. Intra-arterial administration offers the pharmacokinetic advantage of delivering the fibrinolytic agent directly into the treated vascular territory, thereby increasing local drug exposure at the presumed site of residual distal thromboembolism while limiting systemic exposure relative to intravenous administration. The CHOICE trial provided the first randomized evidence that this strategy could translate into improved 90-day functional outcome, using intra-arterial alteplase—recombinant tissue-type plasminogen activator (tPA)—at a dose of 0.225 mg/kg [4].

Eight further randomized trials have since tested variations in this principle, and the resulting pharmacologic heterogeneity is considerable [5,6,7,8,9,10,11,12]. Three fibrinolytic agents have been evaluated: alteplase, a first-generation recombinant tPA requiring prolonged infusion because of its short plasma half-life; tenecteplase, a bioengineered tPA variant bearing substitutions that confer greater fibrin specificity, resistance to plasminogen activator inhibitor-1, and a substantially longer half-life permitting single-bolus administration; and urokinase, a non-fibrin-specific serine protease that activates plasminogen independent of fibrin binding and remains in clinical use in China because of its accessibility and affordability [7]. Dosing strategies likewise varied substantially across the randomized evidence base, although direct numerical comparison across fibrinolytic agents is not pharmacologically meaningful. Tenecteplase was evaluated at 0.0313, 0.0625, and 0.125 mg/kg, alteplase at 0.225 mg/kg, and urokinase as a 100,000-IU intra-arterial dose. This pharmacological heterogeneity was accompanied by important differences in trial context, including vascular territory-predominantly anterior circulation, with basilar artery occlusion studied specifically in IAT-TOP- and the post-thrombectomy reperfusion threshold required for enrollment.

Several conventional and network meta-analyses have examined this evolving literature, including more recent syntheses addressing comparative effects across fibrinolytic agents and dosing regimens [13,14]. For the present cumulative reassessment, two earlier conventional syntheses are particularly informative because their search periods preceded the most recent randomized evidence and therefore provide a clinically relevant reference for examining subsequent changes in the benefit–risk profile. Palaiodimou and colleagues, pooling seven trials and 2131 patients through a March 2025 search, reported that adjunctive IAT increased the likelihood of excellent functional outcome (risk ratio [RR] 1.23, 95% confidence interval [CI] 1.11–1.36) without a significant excess of symptomatic intracranial hemorrhage (sICH) or mortality [15]. Doheim and colleagues, incorporating conference abstracts through February 2025, reached a concordant qualitative conclusion using an odds-ratio framework (odds ratio 1.47, 95% CI 1.21–1.80) [16]. Taken together, these two syntheses supported a consistent interpretation that adjunctive IAT improves excellent functional recovery without a statistically significant excess of sICH or mortality [15,16]. That impression necessarily predates the two major randomized trials published subsequently: IAT-TOP, which extended the evidence base specifically to patients with basilar artery occlusion, and CHOICE-2, a 440-patient trial in which 90-day mortality was 12.1% with adjunctive alteplase and 6.4% with thrombectomy alone. Neither finding could have informed the earlier syntheses because both trials became available only after their respective searches had closed.

This is the gap the present study addresses, and it is deliberately narrower than the question two concordant prior syntheses have already answered. We did not undertake to re-demonstrate efficacy. We undertook to determine whether the full, currently available randomized evidence base—re-extracted from primary publications and analyzed under a single harmonized statistical framework—alters the previously reported balance between functional benefit and all-cause mortality. We also sought to identify when any change in that balance emerged as the evidence accumulated. This systematic review and meta-analysis was prospectively registered (PROSPERO CRD420251006577) and conducted in accordance with the Preferred Reporting Items for Systematic Reviews and Meta-Analyses (PRISMA) 2020 statement [17]. To answer the question posed above, we combined outcome-level re-extraction from the final primary trial publications with a cumulative meta-analytic design that traces the pooled estimates for excellent functional recovery and 90-day mortality trial by trial, in the order each result became available—a design chosen specifically to determine whether the change in the mortality profile emerged only after incorporation of CHOICE-2 or instead reflected a pattern already present in the preceding randomized evidence.

## 2. Materials and Methods

### 2.1. Study Design, Protocol, and Rationale for the Updated Evidence Reassessment

This systematic review and meta-analysis were undertaken as an updated reassessment of randomized evidence evaluating adjunctive IAT administered after successful or near-successful EVT for acute ischemic stroke. The review was conducted in accordance with the PRISMA 2020 statement and was prospectively registered in PROSPERO (CRD420251006577) [17].

The original evidence search was completed on 7 March 2025. Following the subsequent publication of additional randomized trials, the review was reopened and the evidence base was re-extracted and re-analyzed rather than simply extending the numerical results of the earlier six-trial dataset. The updated methodological framework incorporated newly eligible randomized evidence, a paired reassessment of excellent functional recovery and 90-day all-cause mortality, trial-exclusion sensitivity analyses, cumulative meta-analysis, meta-analytic fragility and reverse-fragility analyses, RoB 2 assessment, and outcome-specific evaluation of certainty of evidence using GRADE. Methodological amendments introduced for the updated evidence reassessment were documented in subsequent versions of the registered protocol. Because these amendments were formalized after completion of the updated analyses, they are distinguished from the prospectively registered methods. For the updated analyses, RR was selected as the common relative effect measure for binary efficacy and safety outcomes, permitting evaluation on a consistent relative scale across trials. Random-effects synthesis used REML estimation with Hartung–Knapp inference to account for between-study heterogeneity and uncertainty in the pooled estimates.

The rationale for the present reassessment was not to establish efficacy de novo. Previous synthesis of the randomized evidence had already indicated improved excellent functional recovery with adjunctive IAT without a statistically significant increase in symptomatic intracranial hemorrhage or 90-day mortality [15]. The present review was therefore designed to determine whether this previously reported benefit–risk profile remained consistent as additional randomized evidence accumulated. Cumulative meta-analysis was used to characterize the temporal evolution of the evidence for excellent functional recovery and 90-day all-cause mortality, while the complementary fragility analyses were used to examine the inferential stability of these paired efficacy and mortality findings.

### 2.2. Information Sources and Search Strategy

The original literature search was completed on 7 March 2025. Following the subsequent publication of additional randomized evidence, the search was reopened and updated through 15 August 2026. The updated search was designed to identify potentially eligible studies evaluating adjunctive intra-arterial fibrinolytic therapy administered after successful or near-successful endovascular reperfusion for acute ischemic stroke. No restriction was imposed according to efficacy or safety outcome; study eligibility was therefore independent of the modified Rankin Scale (mRS) threshold reported, mortality, sICH, or other clinical endpoints. PubMed/MEDLINE, Embase, and the Cochrane Central Register of Controlled Trials (CENTRAL) were searched using database-appropriate combinations of controlled vocabulary and free-text terms encompassing acute ischemic stroke or cerebral infarction, endovascular thrombectomy or endovascular treatment/therapy, intra-arterial/intraarterial thrombolysis or fibrinolysis, and the fibrinolytic agents alteplase, tenecteplase, and urokinase.

The complete reproducible search strategies for PubMed/MEDLINE, Embase, and CENTRAL are provided in the Supplementary Materials. The electronic search was supplemented by backward and forward citation tracking of eligible randomized trials, associated protocols and secondary reports, and relevant systematic reviews and meta-analyses. No language restrictions were applied. Records retrieved from the bibliographic databases were deduplicated before screening. For the expanded CENTRAL and Embase searches, deduplicated records underwent rule-based automated prescreening to remove records lacking the intervention concept or representing reviews or editorial material; records retained after prescreening underwent title and abstract assessment. PubMed/MEDLINE records underwent title and abstract screening as in the original review. Potentially eligible reports were subsequently assessed in full text. Multiple publications arising from the same randomized population were linked to their corresponding parent trial. Protocols, corrections, and secondary analyses were retained as companion reports when relevant to study identification, data verification, or risk-of-bias assessment, but were not counted as independent randomized trials.

ClinicalTrials.gov was searched separately through 15 August 2026, and trial-registry records retrieved through the CENTRAL and Embase searches were also assessed to identify ongoing, terminated, or completed-but-unpublished randomized studies relevant to the review question for which analyzable outcome data were unavailable at search closure. Registry records were assessed at the study level and cross-referenced with bibliographic records, protocols, and primary reports to prevent duplicate counting of the same randomized study. After parent-study linkage and deduplication, eight unique randomized studies without analyzable published results at the search-closure date were retained in the systematic-review record. Registered studies without analyzable published results were retained within the systematic review record but did not contribute to quantitative synthesis.

Eligibility for the systematic review and eligibility for an individual quantitative synthesis were considered separately. A randomized trial could therefore remain eligible for the review while not contributing to a particular meta-analysis when the numerical information required to derive the effect measure specified for the updated analysis was unavailable. Missing event counts were not reconstructed from rounded percentages, secondary reports, or graphical inspection.

**Figure 1:**
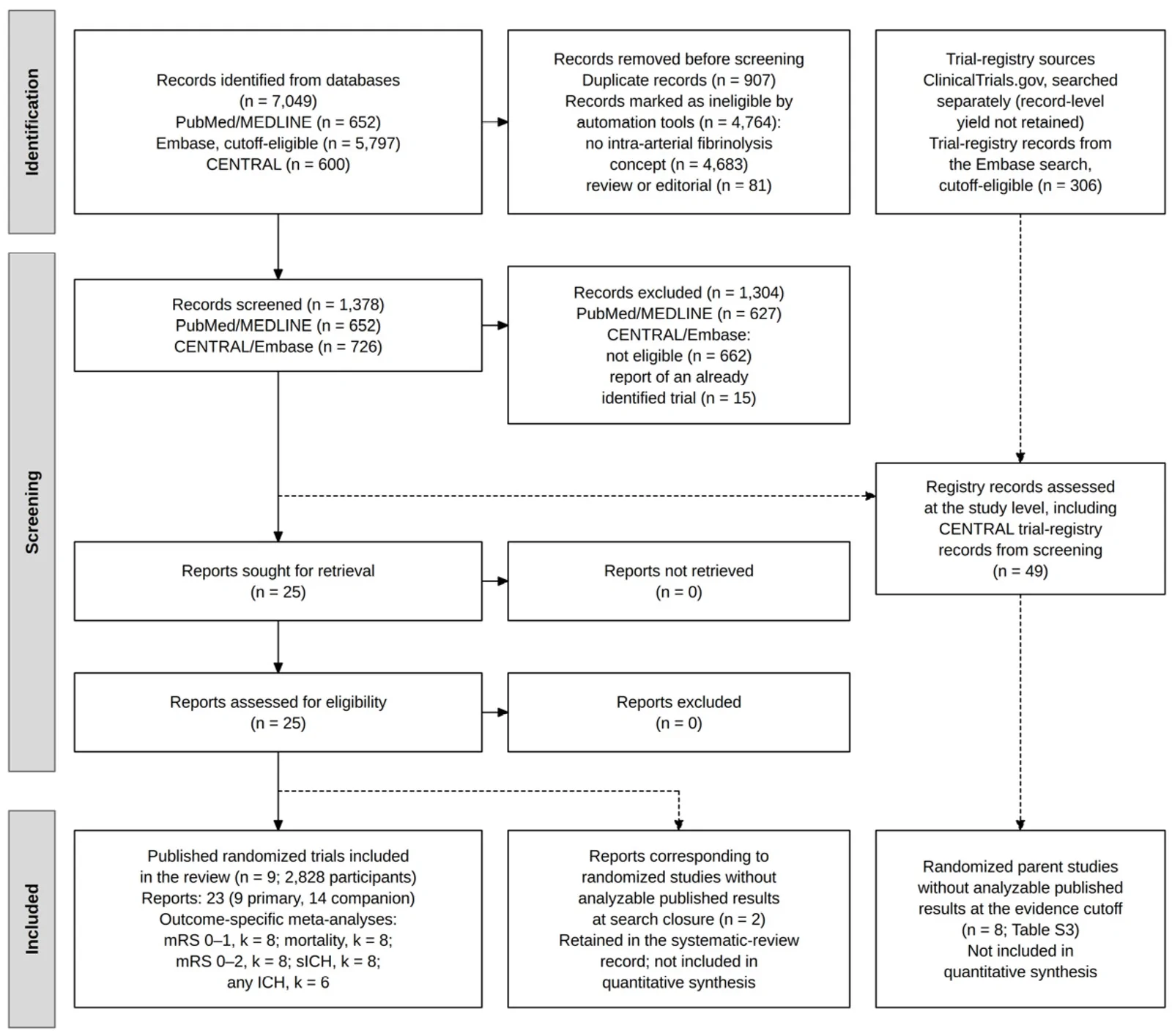
PRISMA 2020 flow diagram of study identification and selection. The updated PubMed/MEDLINE search identified 652 unique records. CENTRAL yielded 600 records, and the cutoff-eligible Embase bibliographic search yielded 5797 records. Across PubMed/MEDLINE, CENTRAL, and Embase, 7049 bibliographic records were identified; removal of 907 duplicate records left 6142 unique records. Rule-based automated prescreening of the CENTRAL/Embase-only records marked 4764 records as ineligible, leaving 1378 records for title and abstract screening across the three databases. Of these, 652 were PubMed/MEDLINE records and 726 were unique CENTRAL/Embase records. Among the latter, 662 were excluded after record-level assessment, 15 were linked to already identified randomized-trial families and were not counted as additional reports, and 49 registry records proceeded to study-level registry assessment. Screening of the additional CENTRAL and Embase records identified no further published randomized trial meeting the prespecified eligibility criteria for quantitative synthesis. In the PubMed/MEDLINE pathway, 25 reports were assessed for eligibility. Twenty-three reports were linked to nine independent published randomized trials, comprising nine primary trial reports and 14 companion reports arising from the same randomized studies. Two additional PubMed-indexed reports corresponded to randomized studies without analyzable published outcome results at the search-closure date; these reports were retained in the systematic-review record but did not contribute outcome data to quantitative synthesis. ClinicalTrials.gov was searched separately, and trial-registry records retrieved through the CENTRAL and Embase searches were also assessed for randomized evidence without analyzable published results. After study-level linkage and deduplication, eight unique randomized parent studies without analyzable published results at the search-closure date were retained in the systematic-review record; registry records were not combined with the bibliographic report-level counts shown in the flow diagram. Dashed connectors distinguish evidence retained for systematic-review documentation from the report-level selection pathway contributing to quantitative synthesis.

The overall study-selection process is summarized in the PRISMA 2020 flow diagram (Figure 1) [17].

### 2.3. Eligibility Criteria

Eligible studies were randomized controlled trials enrolling adults with acute ischemic stroke in the endovascular reperfusion setting who had achieved the angiographic reperfusion threshold required by the individual trial before randomization. The intervention of interest was adjunctive intra-arterial fibrinolytic therapy administered after this post-EVT reperfusion endpoint, compared to a concurrent randomized control group receiving no adjunctive intra-arterial fibrinolytic therapy. Eligibility additionally required reporting of at least one clinically relevant efficacy or safety outcome.

Trials evaluating alteplase, tenecteplase, or urokinase were eligible regardless of fibrinolytic dose, vascular territory, or previous intravenous thrombolysis. Because the angiographic threshold defining eligibility for post-reperfusion treatment was not identical across trials, each study’s original reperfusion criterion was preserved rather than retrospectively recoded to a uniform threshold.

Studies were excluded when treatment allocation was nonrandomized or when the design was observational, single-arm, or case-series. Trials evaluating intravenous thrombolysis alone were outside the scope of the review, as were studies in which intra-arterial fibrinolysis was administered as rescue therapy before achievement of the trial-defined post-EVT reperfusion threshold. When a study incorporated both randomized and nonrandomized phases, only the randomized component was eligible for quantitative synthesis.

### 2.4. Study-Level and Outcome-Level Eligibility

The randomized evidence base comprised CHOICE, ATTENTION-IA, POST-TNK, POST-UK, ANGEL-TNK, DATE, PEARL, IAT-TOP, and CHOICE-2 [4,5,6,7,8,9,10,11,12]. DATE incorporated sequential phase 1b and phase 2a components; in accordance with the eligibility criteria, only its randomized phase 2a component entered the randomized evidence synthesis, whereas the preceding nonrandomized phase 1b dose-escalation component was excluded [9].

Study-level eligibility and eligibility for quantitative synthesis were determined separately. A randomized trial satisfying the clinical and methodological eligibility criteria remained part of the systematic review irrespective of whether numerical data were available for every prespecified outcome. Contribution to an individual meta-analysis required sufficient numerical information to derive the effect measure specified for the updated analysis from the analysis population reported by the trial.

Quantitative extraction was restricted to numerical data reported in the final primary publication or official supplementary material. Raw event counts were not reconstructed from rounded percentages, inferred from graphical displays, or substituted from secondary publications when source-verifiable data were unavailable. When an eligible trial did not provide the numerical information required for a particular outcome, that outcome was not incorporated into the corresponding quantitative synthesis, and no imputation was undertaken. Investigators were not contacted to obtain unpublished outcome data; accordingly, quantitative synthesis was confined to numerical information that could be verified directly from the final primary publications or their official supplementary materials.

### 2.5. Data Extraction and Source Verification

All trial characteristics and quantitative outcome data were re-extracted from final primary trial publications and, when required, official supplementary material. Numerical results were not carried forward solely from the previous manuscript, earlier meta-analyses, conference reports, press releases, or other secondary sources.

For each trial, the extracted variables comprised study design, country and participating centers, randomized sample size, analysis population, vascular territory and qualifying occlusion site, treatment time window, reperfusion criterion at randomization, previous intravenous thrombolysis, fibrinolytic agent, dose and mode of administration, primary endpoint, outcome definitions and ascertainment windows, and outcome-specific event counts and denominators.

Every event/total pair entering a quantitative synthesis was required to be traceable to the final primary publication or official supplementary material. Rounded percentages were not reverse-engineered into integer event counts. When an outcome was reported only as a rounded percentage or adjusted effect estimate without an explicit raw numerator and denominator, it was not included in a raw-event meta-analysis.

Internal inconsistencies within primary reports were documented rather than silently reconciled. In ATTENTION-IA, the primary report gave 8 versus 3 sICH events among 104 participants in each randomized group; the corresponding unadjusted RR of 2.67 reproduces the event-count comparison, whereas the percentages printed alongside those counts do not correspond exactly to denominators of 104 [5]. The explicit event counts and reported analysis population were therefore retained. No clarification of this discrepancy was sought from the ATTENTION-IA investigators. Study selection, data extraction, source verification, and methodological assessments were performed independently by two reviewers (B.Y. and L.C.O.); the study-selection process for the expanded CENTRAL and Embase searches additionally incorporated the rule-based automated prescreening described in Section 2.2. Disagreements were resolved initially through discussion between the two reviewers; when consensus could not be reached, the issue was referred to the senior authors (I.Y. and E.I.C.) for adjudication and resolution by consensus.

### 2.6. Analysis Populations

The included trials did not use identical analysis populations for all outcomes. CHOICE and CHOICE-2 reported principal outcomes using treated-as-randomized or modified analysis populations after a small number of post-randomization exclusions, whereas the remaining trials generally used intention-to-treat or full-analysis-set populations with no or minimal post-randomization exclusion [4,5,6,7,8,9,10,11,12].

Outcome denominators were retained as reported in the final primary publications. Participants excluded from a trial’s reported analysis population were not reintroduced through imputation, and denominators were not artificially harmonized across studies.

Differences in analysis population were incorporated into the RoB 2 assessment and examined through sensitivity analyses excluding CHOICE and CHOICE-2 separately. These analyses were used to assess the robustness of the pooled estimates to post-randomization exclusions and were not interpreted as implying that one analysis population was intrinsically valid and another invalid.

### 2.7. Outcomes

The updated evidence reassessment focused on two paired benefit–safety outcomes: excellent functional recovery, defined as mRS 0–1 at 90 days, and all-cause mortality at 90 days.

Key secondary outcomes were functional independence, defined as mRS 0–2 at 90 days; sICH; any intracranial hemorrhage (ICH); and the ordinal distribution of mRS when a statistically compatible effect measure or sufficiently complete category-level data were available.

The mRS 0–1 and mRS 0–2 endpoints were treated as distinct but nested functional thresholds. Because both arise from the same ordinal scale and are evaluated in overlapping participants, their pooled estimates are statistically dependent. The two pooled RRs were therefore not formally contrasted as though they represented independent treatment groups, and differences in statistical significance between the thresholds were not interpreted as evidence of a statistically significant difference between treatment effects.

### 2.8. Hemorrhagic Outcomes

Definitions and ascertainment windows for hemorrhagic outcomes were extracted separately for each trial.

Definitions of sICH were not uniform and included modified Heidelberg criteria, ECASS III criteria, and trial-specific definitions incorporating neurological deterioration associated with intracranial hemorrhage. Assessment windows varied across trials. Statistical homogeneity of the pooled sICH estimate was therefore not regarded as evidence of clinical equivalence among the underlying hemorrhagic definitions.

The construct designated any ICH was less uniform still and encompassed any cerebral hemorrhage, radiologically detected ICH, and hemorrhage assessed within differing post-treatment windows. CHOICE-2 reported any asymptomatic intracranial hemorrhage; this endpoint was not relabelled as total any ICH and was not entered into the any-ICH synthesis because the two constructs are not equivalent.

Differences in hemorrhagic outcome definition and ascertainment were incorporated into the assessment of indirectness and certainty of evidence.

### 2.9. Handling of the Multi-Arm DATE Trial

The randomized phase 2a component of DATE included two intra-arterial tenecteplase groups, receiving 0.0313 mg/kg and 0.0625 mg/kg, respectively, with a single shared control group [9].

For the overall pairwise meta-analysis, the two randomized tenecteplase groups were combined into a single intervention group for each binary endpoint, and the shared comparator was counted once. This approach avoided duplicate inclusion of the common control group and the resulting unit-of-analysis error, consistent with Cochrane guidance for multi-arm randomized trials [18].

The nonrandomized phase 1b component was excluded from all pooled randomized analyses. A DATE-excluded sensitivity analysis was additionally undertaken to evaluate whether inclusion of this early-phase, multi-arm randomized component materially influenced the overall estimates.

### 2.10. Ordinal mRS Outcomes

A single pooled ordinal mRS estimate was not imposed across the eligible trials because the reported ordinal estimands were not uniform.

The trials variously reported proportional-odds common ORs, generalized ORs derived from Mann–Whitney-type analyses, dose-specific generalized ORs, or ordinal tests without an extractable common effect estimate. ATTENTION-IA and IAT-TOP provided adjusted ordinal ORs; POST-TNK and POST-UK reported generalized ORs; ANGEL-TNK used a Wilcoxon–Mann–Whitney framework; DATE reported dose-specific generalized ORs without a valid published estimate for the combined randomized tenecteplase groups; and CHOICE-2 reported an ordinal analysis without an extractable common OR in the report used for extraction.

These measures were not assumed to represent interchangeable estimands merely because they described the same ordinal clinical scale. Combining statistically non-equivalent estimands would yield a pooled quantity of uncertain interpretation. Ordinal outcomes were therefore eligible for quantitative synthesis only when a common estimand or sufficiently complete category-level data permitted analysis under a common statistical model; otherwise, trial-level ordinal findings were retained for descriptive synthesis. This approach was consistent with Cochrane principles requiring clinical and statistical compatibility of effect estimates before quantitative pooling [18].

### 2.11. Risk-of-Bias Assessment

Risk of bias was assessed using the revised Cochrane Risk of Bias tool for randomized trials (RoB 2) [19]. RoB 2 assessments were performed independently by two reviewers (B.Y. and L.C.O.). Disagreements were resolved through discussion; when consensus could not be reached, the issue was referred to the senior authors (I.Y. and E.I.C.) for adjudication and resolution by consensus. Five domains were considered: bias arising from the randomization process; bias due to deviations from intended interventions; bias due to missing outcome data; bias in measurement of the outcome; and bias in selection of the reported result.

Judgments were informed, where available, by the final primary report, protocol, statistical analysis plan, and trial-registration record. Each domain and the overall judgment were classified as low risk, some concerns, or high risk. Post-randomization exclusions, discrepancies between planned and achieved sample size, early trial termination, and limitations in outcome-reporting availability were considered within the relevant RoB 2 domains rather than treated as automatic evidence of bias.

### 2.12. Certainty of Evidence

Certainty of evidence was assessed separately for each prespecified outcome included in quantitative synthesis using the GRADE framework [20]. The domains considered were risk of bias, inconsistency, indirectness, imprecision, and publication or reporting bias.

GRADE judgments were outcome-specific. Variation in sICH and any-ICH definitions and ascertainment windows was considered under indirectness rather than inferred solely from statistical heterogeneity measures such as I^2^. Sparse event counts and confidence intervals compatible with clinically important benefit and harm were considered under imprecision. Absence of statistical heterogeneity or formal evidence of small-study effects was not, by itself, interpreted as evidence of high certainty.

### 2.13. Statistical Analysis

Binary outcomes were summarized as risk ratios (RRs) with 95% confidence intervals (CIs), calculated from the event counts and denominators reported for the corresponding trial analysis populations. RR was used consistently as the principal relative effect measure for dichotomous efficacy and safety outcomes.

The primary meta-analytic approach was a random-effects model, with between-study variance (τ^2^) estimated using restricted maximum likelihood (REML). Confidence intervals around the pooled mean effect were calculated using the Hartung–Knapp method [21]. Statistical heterogeneity was characterized using Cochran’s Q, I^2^, and τ^2^. I^2^ and REML τ^2^ were interpreted as complementary rather than interchangeable measures of heterogeneity; consequently, an I^2^ estimate at its lower boundary of 0% was not assumed to require an exactly zero REML estimate of τ^2^.

For outcome syntheses including at least five studies, 95% prediction intervals were calculated to complement the pooled mean effect and characterize the range within which the underlying effect of a future comparable study might plausibly lie [22]. Confidence intervals for the pooled mean effect were based on Hartung–Knapp uncertainty, whereas prediction intervals followed the Higgins–Thompson–Spiegelhalter convention specified for the updated analysis, using the classical random-effects standard error combined with τ^2^ and a t distribution with k − 2 degrees of freedom. Prediction intervals were not reported for syntheses containing fewer than five studies because of their limited stability and interpretability with very small numbers of trials [18,22].

Sparse binary outcomes were handled using a treatment-arm continuity-correction strategy selected to account for unequal randomized group sizes. When a study contained a zero-event arm but not zero events in both arms, the primary inverse-variance analysis used a treatment-arm continuity correction accounting for unequal randomized group sizes. This strategy was adopted because constant continuity corrections may distort relative-effect estimates, particularly when treatment groups are unequal in size [23]. Trials with zero events in both groups, if encountered, were not considered informative for estimation of a relative RR and were described separately.

Additional sensitivity analyses excluded CHOICE because the trial was terminated before its intended enrollment, DATE to assess the influence of its early-phase multi-arm randomized design, and CHOICE-2 to characterize the evidence base before incorporation of the most recent large alteplase trial. Sensitivity analyses were used to assess robustness and were not used to select the model producing the most favorable estimate.

Cumulative meta-analyses were performed for excellent functional recovery and 90-day all-cause mortality. Trials were ordered according to their earliest online publication date. When two trials were first published on the same date, they entered the cumulative evidence base simultaneously as a publication-date block, thereby avoiding arbitrary within-day ordering. The first cumulative step, containing a single study, was represented by that study’s conventional log-RR estimate and normal-approximation 95% CI because between-study variance and Hartung–Knapp inference are not estimable with k = 1. From the first multi-study cumulative step onward, the updated REML/Hartung–Knapp framework was applied. Cumulative analyses were used to characterize the temporal evolution and stability of the randomized evidence and were not interpreted as formal sequential monitoring tests.

All statistical analyses were performed using R version 4.6.0 (R Foundation for Statistical Computing, Vienna, Austria) under 64-bit Windows 11. The analytical workflow was implemented using fully reproducible base-R scripts, without reliance on external statistical packages for estimation. The primary analytical framework incorporated inverse-variance random-effects pooling, REML estimation of between-study variance, Hartung–Knapp inference, prediction intervals, sparse-event handling, trial-exclusion sensitivity analyses, and cumulative meta-analysis. Fragility analyses were implemented within the same effect-measure and inferential framework, with the complete meta-analysis re-estimated after every admissible event-status modification. For the mortality analysis, both the conventional iterative pathway and the boundary-optimized bidirectional search were retained as reproducibility records, together with the candidate-state audit trails used to verify the significance-transition states. The finalized outcome-level dataset, analysis scripts, numerical outputs, audit records, and session information were retained to permit independent verification of the analytical workflow.

### 2.14. Subgroup and Moderator Analyses

Formal subgroup analyses were not performed because the number and distribution of randomized trials were insufficient to support clinically and statistically interpretable assessments of effect modification according to fibrinolytic agent, dose, vascular territory, reperfusion context, or previous intravenous thrombolysis (IVT). These characteristics were also strongly correlated at the trial level; consequently, comparisons across such subgroups would largely have contrasted different trials rather than randomized treatment strategies.

Meta-regression was not performed for the same reason. With only nine eligible randomized trials and multiple correlated candidate moderators, study-level meta-regression would have been vulnerable to unstable coefficient estimation, ecological bias, and confounding among trial characteristics. Cochrane guidance cautions against overinterpretation of meta-regression when few studies are available [18].

Accordingly, no inference regarding differential treatment effects according to fibrinolytic agent, dose, vascular territory, reperfusion threshold, or previous IVT was derived from cross-trial comparisons in the present meta-analysis.

### 2.15. Small-Study Effects and Missing-Evidence Assessment

Formal tests of funnel–plot asymmetry, including Egger’s regression test, were not performed because no outcome-specific meta-analysis contained 10 or more studies. With fewer studies, such tests have limited power to distinguish genuine small-study effects from chance and may provide misleading evidence either for or against asymmetry [18,24]. This decision was based on the size of the available evidence base and was independent of the direction or statistical significance of the pooled estimates.

The absence of formal funnel–plot asymmetry testing was not interpreted as evidence that publication bias or other forms of missing-evidence bias were absent. Potential bias arising from unavailable or selectively reported evidence was instead assessed at the outcome level by considering trial registrations and protocols, completeness of outcome reporting, availability of prespecified endpoints, and the extent to which eligible randomized trials could contribute to each quantitative synthesis.

Eligible trials for which the numerical data required for a particular outcome were unavailable remained part of the systematic review but were not included in that outcome-specific meta-analysis; missing values were not imputed. Outcome-level nonavailability was considered, where applicable, in RoB 2 judgments concerning selection of the reported result and in the GRADE assessment of publication or reporting bias.

### 2.16. Trial Sequential Analysis and Sequential Interpretation of the Evidence

Trial sequential analysis (TSA) was considered but was not performed. TSA requires prespecification of assumptions governing the required information size and sequential monitoring boundaries, including a clinically meaningful intervention effect and acceptable type I and type II error rates. For 90-day mortality, no externally established and clinically justified relative-effect threshold was identified that could be adopted prospectively without introducing an arbitrary assumption.

Accordingly, trial-sequential monitoring boundaries were not derived from the accumulating trial results, and no anticipated intervention effect was selected retrospectively from the observed evidence. Instead, the temporal evolution of the randomized evidence was evaluated using cumulative meta-analysis, with trials ordered according to their earliest online publication date and trials first published on the same date entering simultaneously as a publication-date block.

Cumulative meta-analysis was used to characterize the evolution of pooled effect estimates and their uncertainty as randomized evidence accumulated. It was not interpreted as a formal sequential test of benefit, harm, or futility. Inferential conclusions were based on the updated meta-analytic framework, including pooled effect estimates and their precision, between-study heterogeneity, prediction intervals where applicable, sensitivity analyses, risk-of-bias assessments, and GRADE certainty of evidence.

### 2.17. Fragility Analysis

To complement conventional measures of effect magnitude and statistical uncertainty, meta-analytic fragility was examined for the two principal paired efficacy–safety outcomes: excellent functional recovery (mRS 0–1 at 90 days) and 90-day all-cause mortality. For the statistically significant mRS 0–1 synthesis, the meta-analytic Fragility Index (FI) was evaluated according to the iterative event-status modification procedure described by Atal et al. [25]. At each step, all admissible one-patient changes in event status across the contributing treatment and control arms were considered, and the modification moving the relevant confidence-interval boundary furthest toward the null was retained. The FI was defined as the number of sequential modifications required under this procedure for the pooled effect to lose statistical significance.

Mortality required a different inferential approach because the primary pooled estimate was not statistically significant and a reverse fragility analysis must accommodate transitions in either direction. Accordingly, pathways toward both increased and reduced mortality with adjunctive IAT were evaluated in parallel. An initial Atal-style iterative analysis was complemented by a boundary-optimized search, recognizing that sequential selection of the locally most influential one-patient modification need not identify the earliest significance-transition state once the pooled effect, between-study variance, and confidence interval are re-estimated after every change. In the direction of increased mortality, admissible modifications comprised the addition of mortality events to the adjunctive-IAT arm or their removal from the control arm, and candidate states were prioritized according to the lower 95% confidence-interval boundary. The converse modifications were evaluated in the direction of reduced mortality and prioritized according to the upper confidence-interval boundary. The boundary-optimized search used a deterministic finite-width candidate strategy and proceeded through 24 modifications in each direction. At each modification depth, up to 8000 candidate states were retained, and up to 2500 prioritized candidates per direction underwent exact re-estimation using the complete meta-analytic model; at depth 24 in the harm direction, the separately verified crossing state was additionally evaluated. The earliest verified crossing of the significance boundary determined the direction of reverse fragility.

After every event-status modification, the meta-analysis was re-estimated de novo using the same effect measure and inferential framework as the primary analysis: risk ratios, restricted maximum likelihood estimation of between-study variance, and Hartung–Knapp confidence intervals. Statistical significance was defined by a two-sided α level of 0.05 and, equivalently, by exclusion of RR = 1 from the 95% confidence interval. Single-zero-arm states arising during the mortality search were handled using the same treatment-arm continuity-correction convention applied to sparse-event analyses, whereas double-zero trial states were excluded as non-informative for risk-ratio estimation.

Because the boundary-optimized analysis retained a finite number of candidate states rather than exhaustively enumerating every possible event-status configuration, the resulting reverse fragility estimate was interpreted as the earliest significance transition identified by the search and not as proof of a global mathematical minimum. Fragility measures were considered complementary indices of inferential stability rather than measures of treatment-effect magnitude, clinical importance, causality, or certainty of evidence, and were interpreted in conjunction with the pooled estimates, confidence and prediction intervals, sensitivity analyses, risk-of-bias assessments, and GRADE certainty ratings.

### 2.18. Use of Artificial Intelligence

During the preparation of this manuscript, the authors used ChatGPT (OpenAI; GPT-5.5 Thinking) exclusively for English-language editing and grammatical refinement. The tool was not used for any scientific or methodological aspect of the study. All scientific content remains entirely the work of the authors, who reviewed and approved the final manuscript and take full responsibility for its content.

## 3. Results

### 3.1. Study Selection and Randomized Evidence Base

The updated PubMed/MEDLINE search yielded 652 unique records. After title and abstract screening, 627 records were excluded, and 25 reports underwent full eligibility assessment. CENTRAL yielded 600 records, and the cutoff-eligible Embase bibliographic search yielded 5797 records. Across PubMed/MEDLINE, CENTRAL, and Embase, 7049 bibliographic records were identified; removal of 907 duplicate records left 6142 unique records. Rule-based automated prescreening of the CENTRAL/Embase-only records marked 4764 records as ineligible, leaving 1378 records for title and abstract screening across the three databases. Of these, 652 were PubMed/MEDLINE records and 726 were unique CENTRAL/Embase records. Among the latter, 662 were excluded after record-level assessment, 15 were linked to already identified randomized-trial families and were not counted as additional reports, and 49 registry records proceeded to study-level registry assessment. Screening of the additional CENTRAL and Embase records identified no further published randomized trial meeting the prespecified eligibility criteria for quantitative synthesis. Twenty-three reports were linked to nine independent published randomized trials, comprising nine primary trial reports and 14 companion publications arising from the same randomized studies. Two additional PubMed-indexed reports corresponded to randomized studies for which analyzable published outcome results were unavailable at the search-closure date. Study-level assessment of ClinicalTrials.gov and trial-registry records retrieved through the CENTRAL and Embase searches identified eight unique randomized parent studies relevant to adjunctive intra-arterial fibrinolysis for which analyzable published outcome results were unavailable at search closure. These studies did not contribute to quantitative synthesis and are summarized according to their study-level disposition in Table S3. The study-selection process is presented in Figure 1.

The nine included randomized trials were CHOICE, ATTENTION-IA, POST-TNK, POST-UK, ANGEL-TNK, DATE, PEARL, IAT-TOP, and CHOICE-2 [4,5,6,7,8,9,10,11,12], comprising 2828 randomized participants across the systematic-review evidence base. Only the randomized phase 2a component of DATE was included; the preceding nonrandomized phase 1b dose-escalation component was excluded. The principal characteristics of the included trials are summarized in Table 1.

The evidence base encompassed alteplase, tenecteplase, and urokinase regimens; anterior- and posterior-circulation populations; and trial-specific post-EVT reperfusion thresholds ranging from eTICI 2b to eTICI 2c–3.

Outcome availability varied across the nine included trials. Eight trials contributed to the analysis of excellent functional recovery (mRS 0–1 at 90 days), eight to 90-day all-cause mortality, eight to functional independence (mRS 0–2 at 90 days), eight to sICH, and six to the exploratory analysis of any ICH. Outcome-specific trial availability is presented in Table S1.

IAT-TOP contributed to the mRS 0–2 analysis but not to the mRS 0–1, mortality, sICH, or any-ICH syntheses because the raw event counts required for those analyses were unavailable in the source used for extraction. CHOICE-2 contributed to the mRS 0–1, mortality, and sICH analyses but not to the mRS 0–2 or any-ICH syntheses because compatible raw numerical data for those outcomes were unavailable. ATTENTION-IA did not contribute to the any-ICH synthesis because compatible raw total any-ICH data were unavailable. Publications arising from the same randomized population were linked to their parent trial and were not counted as independent studies; companion reports are summarized in Table S2. Study-level assessment of ClinicalTrials.gov and trial-registry records retrieved through the CENTRAL and Embase searches identified eight unique randomized parent studies relevant to adjunctive intra-arterial fibrinolysis for which analyzable published outcome results were unavailable at search closure. These studies did not contribute to quantitative synthesis and are summarized according to their study-level disposition in Table S3.

### 3.2. Risk of Bias

**Figure 2:**
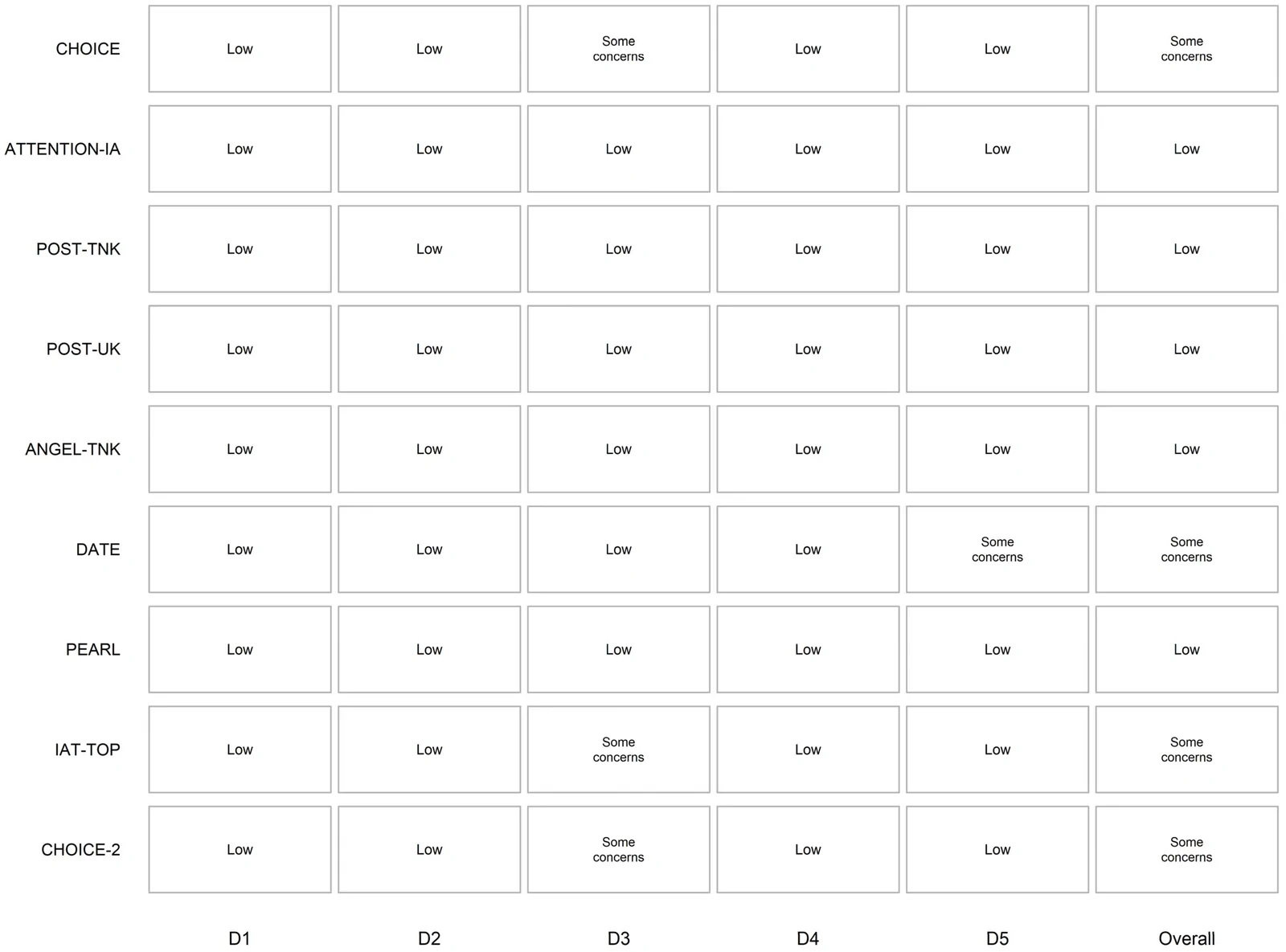
Risk-of-bias assessment of the included randomized trials using the Cochrane RoB 2 tool. Judgments are presented for bias arising from the randomization process (D1), bias due to deviations from intended interventions (D2), bias due to missing outcome data (D3), bias in measurement of the outcome (D4), bias in selection of the reported result (D5), and overall risk of bias.

Five of the nine included randomized trials—ATTENTION-IA, POST-TNK, POST-UK, ANGEL-TNK, and PEARL—were judged to be at low risk of bias overall. CHOICE, DATE, IAT-TOP, and CHOICE-2 were judged as having some concerns, and no trial was judged to be at high risk of bias (Figure 2).

The domain-level concerns were limited to missing outcome data for CHOICE, IAT-TOP, and CHOICE-2 and selection of the reported result for DATE. All nine trials were judged to be at low risk of bias for the randomization process, deviations from intended interventions, and measurement of the outcome.

### 3.3. Excellent Functional Recovery

**Figure 3:**
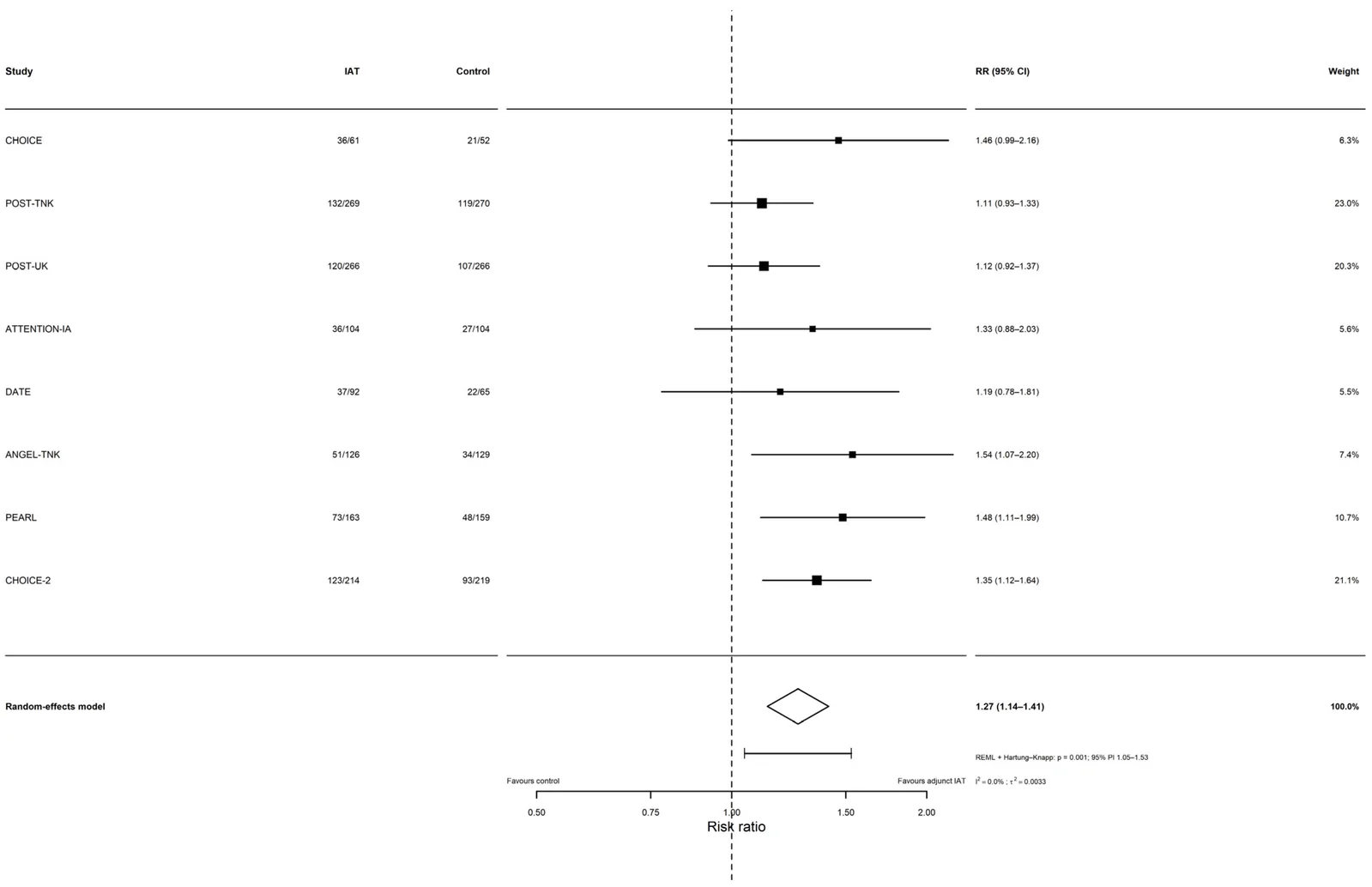
Excellent functional recovery (mRS 0–1) at 90 days. Forest plot comparing adjunctive intra-arterial thrombolysis with control after successful or near-successful endovascular reperfusion. Between-study variance was estimated using restricted maximum likelihood, with Hartung–Knapp inference for the pooled confidence interval. The horizontal interval beneath the pooled estimate represents the 95% prediction interval. Black squares represent individual trial risk-ratio estimates, with horizontal lines indicating their 95% confidence intervals. The diamond represents the pooled random-effects estimate, with its width indicating the corresponding 95% confidence interval. The vertical dashed line indicates the null effect (RR = 1).

Eight randomized trials contributed to the analysis of excellent functional recovery, defined as an mRS score of 0–1 at 90 days. Adjunctive IAT was associated with a significantly higher probability of excellent functional recovery than control (RR 1.266, 95% CI 1.135–1.412; *p* = 0.001) (Figure 3). Between-study heterogeneity was negligible (I^2^ = 0.0%; τ^2^ = 0.0033), and the 95% prediction interval was 1.047–1.530.

### 3.4. Ninety-Day All-Cause Mortality

**Figure 4:**
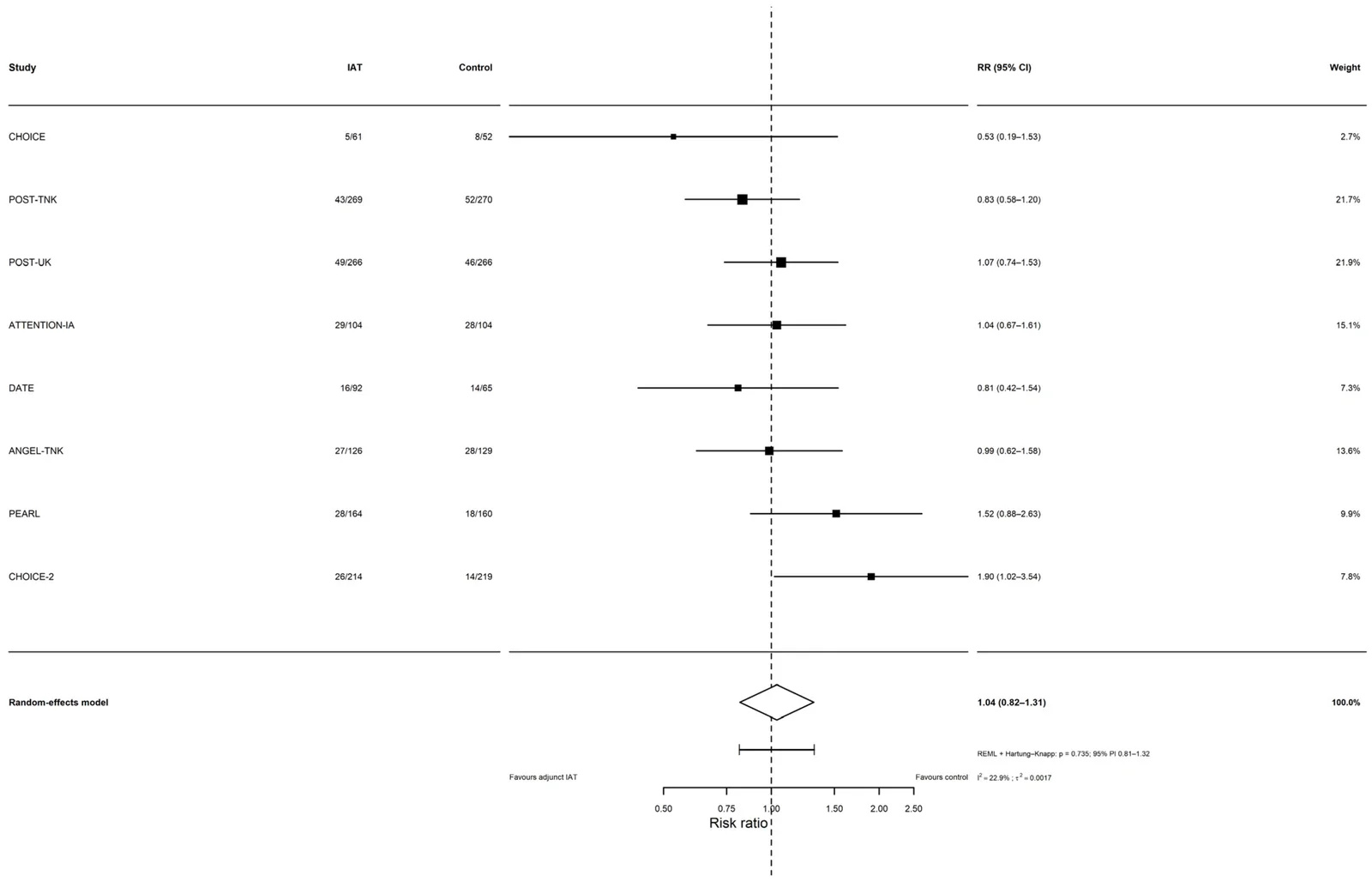
Ninety-day all-cause mortality. Forest plot comparing adjunctive intra-arterial thrombolysis with control after successful or near-successful endovascular reperfusion. Black squares represent individual trial risk-ratio estimates, with horizontal lines indicating their 95% confidence intervals. The diamond represents the pooled random-effects estimate, with its width indicating the corresponding 95% confidence interval. The vertical dashed line indicates the null effect (RR = 1). Between-study variance was estimated using restricted maximum likelihood, with Hartung–Knapp inference for the pooled confidence interval. The horizontal interval beneath the pooled estimate represents the 95% prediction interval.

Eight randomized trials contributed to the analysis of 90-day all-cause mortality. Adjunctive IAT was not associated with a statistically significant difference in mortality compared with control (RR 1.036, 95% CI 0.817–1.314; *p* = 0.735) (Figure 4). Between-study heterogeneity was low (I^2^ = 22.9%), and the 95% prediction interval was 0.815–1.318.

### 3.5. Functional Independence

**Figure 5:**
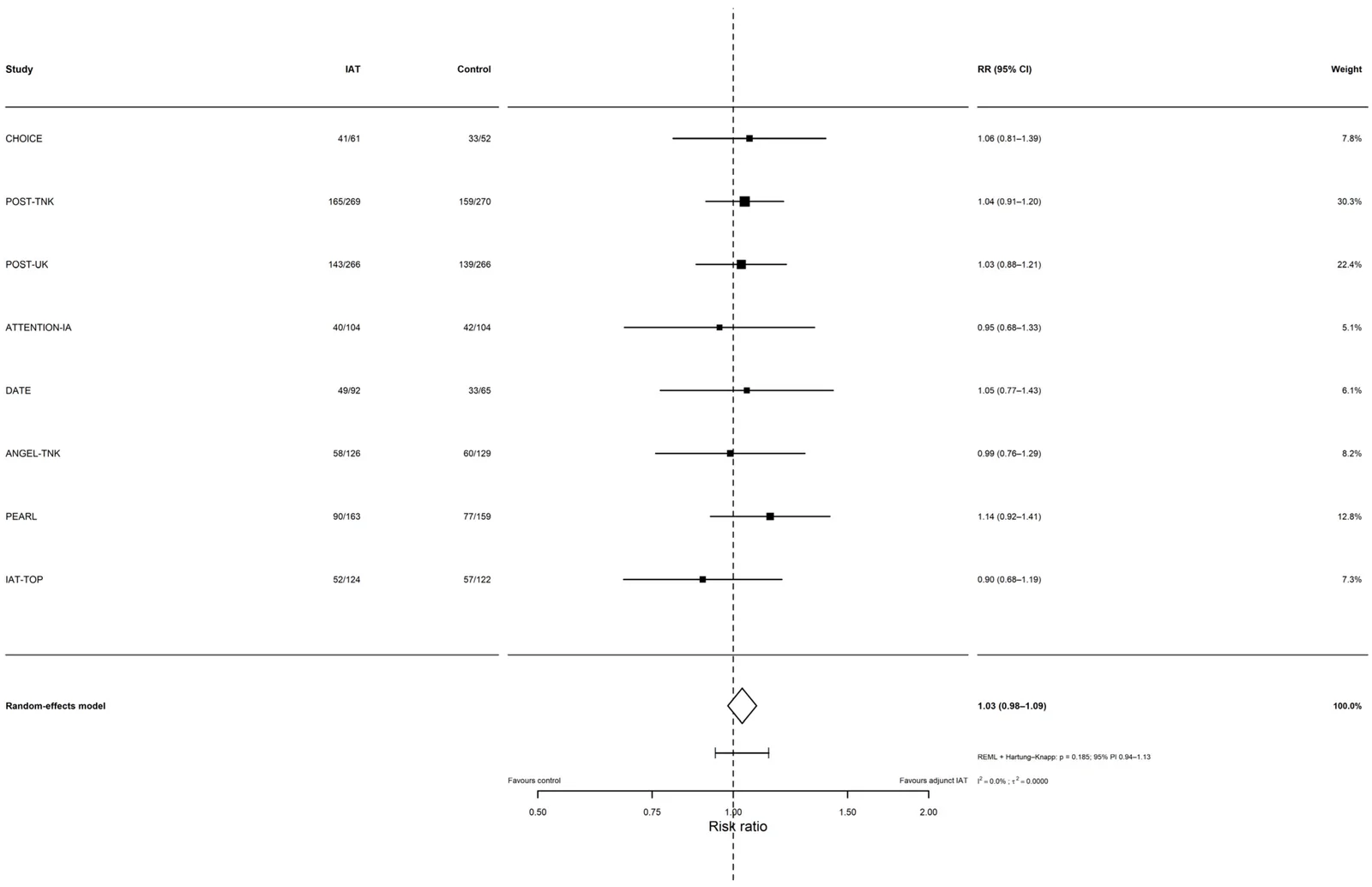
Functional independence (mRS 0–2) at 90 days. Forest plot comparing adjunctive intra-arterial thrombolysis with control after successful or near-successful endovascular reperfusion. Black squares represent individual trial risk-ratio estimates, with horizontal lines indicating their 95% confidence intervals. The diamond represents the pooled random-effects estimate, with its width indicating the corresponding 95% confidence interval. The vertical dashed line indicates the null effect (RR = 1). Between-study variance was estimated using restricted maximum likelihood, with Hartung–Knapp inference for the pooled confidence interval. The horizontal interval beneath the pooled estimate represents the 95% prediction interval.

Eight randomized trials contributed to the analysis of functional independence, defined as an mRS score of 0–2 at 90 days. Adjunctive IAT was not associated with a statistically significant difference in functional independence compared with control (RR 1.032, 95% CI 0.981–1.086; *p* = 0.185) (Figure 5). Between-study heterogeneity was negligible (I^2^ = 0.0%; τ^2^ = 0.0000), and the 95% prediction interval was 0.939–1.135.

### 3.6. Hemorrhagic Safety Results

**Figure 6:**
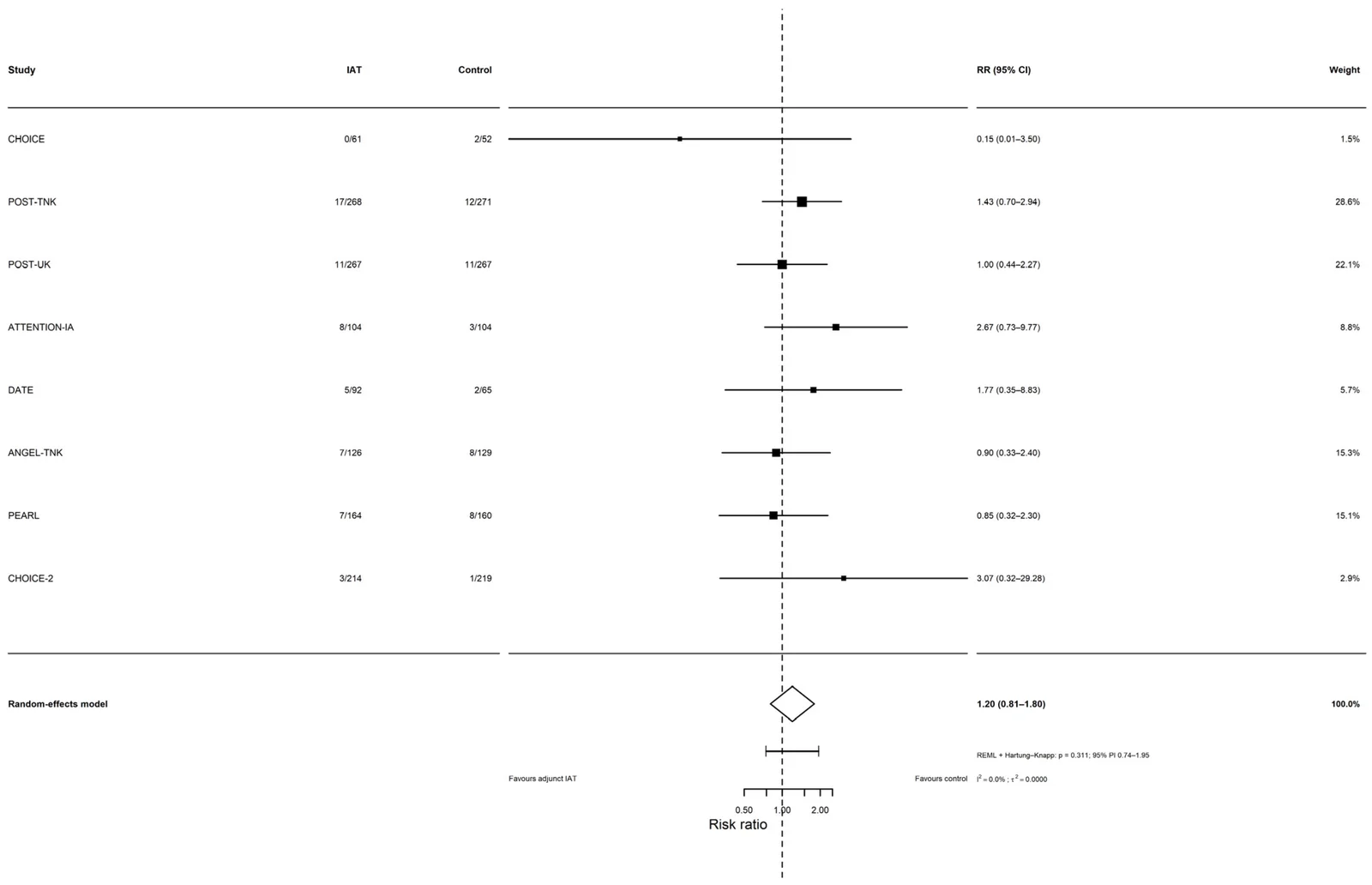
Symptomatic intracranial hemorrhage. Forest plot comparing adjunctive intra-arterial thrombolysis with control. Black squares represent individual trial risk-ratio estimates, with horizontal lines indicating their 95% confidence intervals. The diamond represents the pooled random-effects estimate, with its width indicating the corresponding 95% confidence interval. The vertical dashed line indicates the null effect (RR = 1). Between-study variance was estimated using restricted maximum likelihood, with Hartung–Knapp inference for the pooled confidence interval. The horizontal interval beneath the pooled estimate represents the 95% prediction interval.

Eight randomized trials contributed to the analysis of sICH. Adjunctive IAT was not associated with a statistically significant difference in sICH compared to control (RR 1.203, 95% CI 0.806–1.796; *p* = 0.311) (Figure 6). Between-study heterogeneity was negligible (I^2^ = 0.0%), and the 95% prediction interval was 0.744–1.945. Definitions and ascertainment windows for sICH varied across trials and are summarized in Table S4.

Six randomized trials contributed compatible data to the exploratory analysis of any intracranial hemorrhage. The pooled estimate was RR 1.120 (95% CI 0.938–1.337; *p* = 0.161), with I^2^ = 0.0% and a 95% prediction interval of 0.860–1.458 (Figure S1). ATTENTION-IA, IAT-TOP, and CHOICE-2 did not contribute to this synthesis because compatible total any-ICH data were unavailable.

### 3.7. Ordinal mRS Findings

Ordinal mRS outcomes were reported using heterogeneous statistical estimands across the included trials. CHOICE reported a common OR with exact mRS categories; ATTENTION-IA and IAT-TOP reported adjusted ordinal ORs; POST-TNK and POST-UK reported generalized ORs; ANGEL-TNK used a Wilcoxon–Mann–Whitney framework; DATE reported dose-arm-specific generalized ORs without a valid combined estimate for the two randomized tenecteplase groups; PEARL reported an ordinal OR with exact mRS categories available; and CHOICE-2 reported an ordinal analysis without an extractable common OR in the report used for extraction.

Because these estimands were not statistically equivalent, no overall pooled ordinal mRS estimate was calculated. Trial-specific ordinal estimands and their disposition in the review are presented in Table S5.

### 3.8. Sensitivity Analyses

Trial-exclusion sensitivity analyses were performed for excellent functional recovery and 90-day all-cause mortality (Table S6). For mRS 0–1, exclusion of CHOICE yielded RR 1.254 (95% CI 1.112–1.413; I^2^ = 1.9%; 95% PI 1.023–1.537). Exclusion of DATE yielded RR 1.274 (95% CI 1.125–1.444; I^2^ = 9.9%; 95% PI 1.015–1.600), and exclusion of CHOICE-2 yielded RR 1.246 (95% CI 1.092–1.422; I^2^ = 0.0%; 95% PI 0.993–1.565).

For 90-day all-cause mortality, exclusion of CHOICE yielded RR 1.057 (95% CI 0.827–1.351; I^2^ = 20.1%; 95% PI 0.803–1.391). Exclusion of DATE yielded RR 1.061 (95% CI 0.808–1.394; I^2^ = 29.1%; 95% PI 0.755–1.492), and exclusion of CHOICE-2 yielded RR 0.984 (95% CI 0.801–1.209; I^2^ = 0.0%; 95% PI 0.778–1.245).

### 3.9. Certainty of Evidence and Summary of Findings

The principal pooled estimates and corresponding certainty ratings are summarized in Table 2. Certainty of evidence was rated moderate for excellent functional recovery, 90-day all-cause mortality, and functional independence, and low for sICH and any ICH.

Detailed RoB 2 judgments are presented in Table S7, and the outcome-specific GRADE assessments are presented in Table S8. The GRADE profile incorporated risk of bias, inconsistency, indirectness, imprecision, and publication or reporting bias. Variation in hemorrhagic outcome definitions and ascertainment windows contributed to the certainty assessment for sICH and any ICH.

### 3.10. Cumulative Meta-Analysis

**Figure 7:**
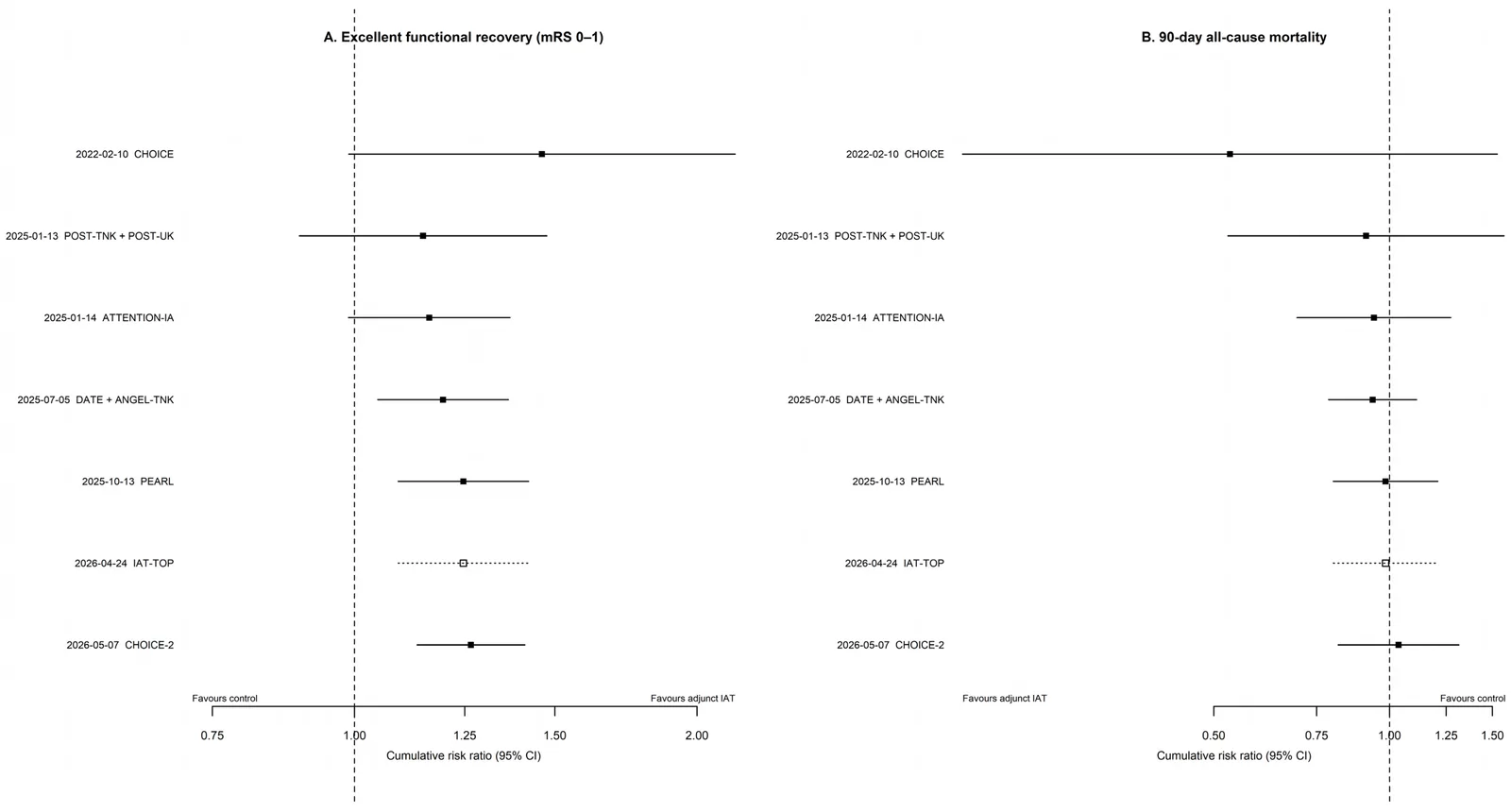
Cumulative meta-analysis according to earliest online publication date. (**A**) Excellent functional recovery (mRS 0–1 at 90 days). (**B**) Ninety-day all-cause mortality. Trials first published on the same date entered the cumulative analysis simultaneously as a publication-date block. The initial CHOICE step is shown as a single-study log-RR estimate with a normal-approximation 95% confidence interval. From the first multi-study step onward, estimates were obtained using the REML random-effects model with Hartung–Knapp confidence intervals. IAT-TOP did not contribute event counts to either cumulative outcome and therefore did not alter the corresponding cumulative estimates at its publication step. Filled black squares represent cumulative risk-ratio estimates, with solid horizontal lines indicating their 95% confidence intervals; the open square and dotted horizontal line at the IAT-TOP step indicate the unchanged estimate and confidence interval carried forward from the preceding publication block. The vertical dashed line indicates the null effect (RR = 1).

Cumulative meta-analysis was performed for excellent functional recovery and 90-day all-cause mortality, with trials ordered according to their earliest online publication date (Figure 7; Table S9).

For mRS 0–1, the cumulative RR was 1.461 (95% CI 0.989–2.160) with CHOICE alone. Following the addition of POST-TNK and POST-UK, the estimate was 1.149 (95% CI 0.895–1.476); after ATTENTION-IA, 1.163 (95% CI 0.988–1.370); after DATE and ANGEL-TNK, 1.196 (95% CI 1.048–1.365); and after PEARL, 1.246 (95% CI 1.092–1.422). IAT-TOP did not contribute mRS 0–1 event counts and therefore did not alter the cumulative estimate. After CHOICE-2, the final cumulative estimate was RR 1.266 (95% CI 1.135–1.412).

For 90-day all-cause mortality, the cumulative RR was 0.533 (95% CI 0.186–1.529) with CHOICE alone, 0.911 (95% CI 0.529–1.570) after POST-TNK and POST-UK, 0.940 (95% CI 0.694–1.273) after ATTENTION-IA, 0.935 (95% CI 0.786–1.113) after DATE and ANGEL-TNK, and 0.984 (95% CI 0.801–1.209) after PEARL. IAT-TOP did not contribute mortality event counts and therefore did not alter the cumulative estimate. After CHOICE-2, the final cumulative estimate was RR 1.036 (95% CI 0.817–1.314).

### 3.11. Fragility Analysis Results

Fragility analysis assessed the statistical stability of the paired efficacy and mortality findings under sequential changes to the observed event data. For excellent functional recovery (mRS 0–1 at 90 days), the meta-analytic Fragility Index was 15. The pooled efficacy signal remained statistically significant through 14 sequential one-patient event-status modifications and crossed the conventional significance boundary after the 15th modification, yielding a pooled RR of 1.219 (95% CI 0.995–1.494; *p* = 0.054).

For 90-day all-cause mortality, the primary pooled estimate was non-significant. In the boundary-optimized bidirectional reverse-fragility analysis, the earliest significance transition identified by the search occurred after 24 one-patient event-status modifications and was in the direction of increased mortality with adjunctive IAT. At this transition, the pooled estimate was RR 1.174 (95% CI 1.002–1.377; *p* = 0.048). No corresponding transition toward reduced mortality was identified through the same modification depth.

These fragility estimates and the corresponding significance-transition states are summarized in Table S10.

## 4. Discussion

The principal finding of this updated and cumulative meta-analysis is not the emergence of a new efficacy signal but the persistence of a previously reported one, set against a safety picture that has grown more complex since the field’s last synthesis. Across eight trials drawn from a total randomized population of 2581 participants, adjunctive IAT was associated with a 26.6% relative increase in the probability of excellent functional recovery (RR 1.266, 95% CI 1.135–1.412), with negligible between-study heterogeneity (I^2^ = 0.0%, τ^2^ = 0.0033) and a 95% prediction interval (1.047–1.530) that did not cross unity. The significance of this estimate is best understood in relation to the earlier syntheses that provide the most relevant temporal reference for the present update, and, more importantly, to the randomized evidence that became available only after their searches had closed.

Palaiodimou and colleagues, working with seven trials through a March 2025 search closure, estimated RR 1.23 (95% CI 1.11–1.36) for the identical outcome [15]. Doheim and colleagues, incorporating conference abstracts through February 2025, arrived at OR 1.47 (95% CI 1.21–1.80) using an odds-ratio framework [16]. The present estimate sits almost precisely between these two figures, and closer to Palaiodimou’s than to Doheim’s. This difference is attributable chiefly to the arithmetic relationship between odds and risk ratios once the control-arm event rate exceeds roughly 30%—the range in which an odds ratio inflates relative to the corresponding risk ratio irrespective of any real difference in the underlying data—rather than to any substantive disagreement between the trial sets. Despite differences in search closure, trial composition, and effect measure, the earlier syntheses and the present analysis converge on a consistent direction and broadly comparable magnitude of benefit for excellent functional recovery. This concordance strengthens the interpretation that the efficacy signal is a reproducible feature of the randomized evidence rather than an artifact of a particular analytical specification. The purpose of the present update was therefore not to re-establish efficacy, but to determine whether that increasingly consistent functional signal has remained accompanied by an equally reassuring mortality profile as the evidence base has matured. This question also has contemporary clinical relevance. The evidence review underpinning the 2026 American Heart Association/American Stroke Association guideline was conducted principally between September and December 2024, with selected high-impact studies incorporated through March 2025; accordingly, several randomized trials now informing the evidence base—including IAT-TOP and CHOICE-2—were not available to that guideline process [26].

Where the present synthesis earns its place is not in the efficacy estimate but in what no previous synthesis of this literature was, by construction, able to examine: CHOICE-2, a 440-patient trial published in May 2026 reporting 90-day all-cause mortality of 12.1% with adjunctive alteplase against 6.4% with thrombectomy alone. This imbalance appears in neither Palaiodimou’s nor Doheim’s synthesis for the simple reason that the trial did not yet exist when their searches closed; it is not a limitation attributable to either group of authors, only a boundary of what a literature review completed in early-to-mid 2025 could contain. The value of the cumulative meta-analytic framework applied here is that it does more than absorb CHOICE-2 into an updated pooled figure—it shows precisely where in the accumulating evidence the picture changed. The pooled mortality estimate remained numerically favorable or close to the null throughout the randomized evidence available before CHOICE-2, standing at RR 0.984 (95% CI 0.801–1.209) immediately before that trial entered the synthesis, a finding broadly consistent with the mortality profile reported in the earlier evidence base. Consistent with this cumulative trajectory, exclusion of CHOICE-2 yielded the same pooled mortality estimate of RR 0.984 (95% CI 0.801–1.209), illustrating the extent to which the current aggregate mortality estimate is influenced by the addition of this single large trial. This should be interpreted neither as a refutation of the earlier reassuring evidence nor as confirmation of treatment-related harm. Rather, it shows that the mortality profile inferred from the earlier randomized evidence is no longer sufficient, by itself, to characterize the expanded evidence base after incorporation of CHOICE-2. Interpretation of the CHOICE-2 mortality imbalance also requires attention to the control-group event rate: 90-day mortality in the control arm was 6.4%, lower than in most other trials contributing mortality data. The aggregate evidence cannot determine whether the observed between-group imbalance reflects a treatment-related effect, trial-specific baseline risk, an unusually low control-group event rate, or some combination of these factors. Resolving these alternatives will require replication or individual-participant-data analyses rather than further re-analysis of the same aggregate event counts.

Fragility analysis adds a distinct dimension to this evolving benefit–risk assessment by quantifying how readily the principal statistical inferences change with sequential changes to the observed event data. The mRS 0–1 efficacy signal persisted through 14 sequential one-patient event-status modifications and crossed the significance boundary only with the 15th, indicating that the principal functional finding was not contingent on one or a few outcome events. This observation complements, rather than substitutes for, the magnitude and precision of the pooled treatment effect: the clinical interpretation of efficacy continues to rest on the RR, its confidence and prediction intervals, and the overall certainty of evidence. Mortality presents a fundamentally different inferential problem. The primary pooled estimate remained non-significant, but the boundary-optimized bidirectional analysis identified its earliest significance transition after 24 one-patient event-status modifications, in the direction of increased mortality with adjunctive IAT (RR 1.174, 95% CI 1.002–1.377; *p* = 0.048). No corresponding transition toward reduced mortality was identified through the same modification depth. This directional asymmetry should not be construed as evidence of treatment-related mortality: fragility analysis interrogates the stability and directionality of a statistical inference, not the causal origin of the underlying events. It does, however, show that the present mortality evidence reaches a significance boundary more readily in the direction of harm than of benefit. Considered together with the cumulative analysis, which localized the change in the pooled mortality estimate to the incorporation of CHOICE-2, these findings sharpen the benefit–risk interpretation without overstating it: the signal for excellent functional recovery is reproducible and comparatively resistant to perturbation of the observed event data, whereas the mortality evidence remains unresolved and does not presently justify a definitive inference of either excess mortality or mortality neutrality.

A second point of divergence concerns functional threshold rather than mortality. Palaiodimou and colleagues additionally reported a shift analysis across the full ordinal mRS distribution, finding a significant reduction in overall disability (common OR 1.10, 95% CI 1.03–1.18) [15]. The present synthesis, constrained to the raw-event mRS 0–2 threshold reported by eight trials, found no significant improvement in functional independence (RR 1.032, 95% CI 0.981–1.086). These are not regarded as contradictory findings so much as differently sensitive instruments applied to the same underlying redistribution: an ordinal shift statistic registers movement anywhere across the disability spectrum, whereas a single dichotomization at mRS 0–2 registers benefit only if a sufficient number of patients cross that specific boundary. Because mRS 0–1 and mRS 0–2 are nested categories evaluated in the same overlapping patients rather than independent endpoints, a treatment effect concentrated at the upper end of the recovery distribution—moving patients from mild disability into the excellent-outcome category rather than from moderate dependency into independence—is entirely compatible with the significant mRS 0–1 result and the null mRS 0–2 result reported side by side in this review. This is regarded as a discordant pattern across prespecified functional thresholds rather than evidence that the mRS 0–1 finding is fragile, and CHOICE-2’s substantial weight in the mRS 0–1 synthesis (21.1%) gives this update statistical purchase on the pattern that the earlier, smaller evidence base did not have.

Posterior-circulation occlusion remains a population the randomized literature has barely begun to characterize. Both Palaiodimou and Doheim had access to only a single such trial, ATTENTION-IA, at the time of their respective searches. The present review adds IAT-TOP, a randomized trial devoted specifically to basilar artery occlusion, though its contribution to this synthesis is partial: raw event counts for mRS 0–1, mortality, and sICH were not extractable from the report used for extraction, limiting IAT-TOP to the mRS 0–2 synthesis, where its result (52/124 versus 57/122) numerically favored the control group. ATTENTION-IA’s own mRS 0–1 estimate (RR 1.33, 95% CI 0.88–2.03) remained compatible with a wide range of true effects. Any inference, from either trial alone or from the two considered together, about whether posterior-circulation physiology modifies the benefit of adjunctive IAT is deliberately avoided. The confidence intervals are too wide and the trials too few, and this is a question the existing randomized literature, including the present synthesis, is simply not yet positioned to answer.

Agent-specific efficacy is a related question deliberately left unexamined here. Of the nine trials assembled here, four evaluated alteplase (CHOICE, PEARL, IAT-TOP, CHOICE-2), four evaluated tenecteplase (ATTENTION-IA, POST-TNK, ANGEL-TNK, DATE), and one evaluated urokinase (POST-UK)—a distribution of fibrinolytic agents skewed enough that a trial-level agent comparison would, in effect, compare trial design rather than drug pharmacology. Doheim and colleagues reported subgroup estimates favoring alteplase (OR 1.94, 95% CI 1.31–2.87) and tenecteplase (OR 1.43, 95% CI 1.08–1.89) but not urokinase (OR 1.22, 95% CI 0.87–1.72) [16]. An equivalent stratification was not attempted here. Fibrinolytic agent is substantially confounded with vascular territory, dose, and trial-level geographic setting across the nine trials assembled here, and a trial-level agent comparison under these conditions would largely contrast different trials rather than different treatment strategies applied to comparable populations. The comparative efficacy of alteplase, tenecteplase, and urokinase is considered unresolved by trial-level randomized evidence from either synthesis, a question that individual-patient-data pooling or a head-to-head randomized comparison could address and trial-level subgroup contrasts cannot. The pooled IAT estimate should therefore be interpreted as an aggregate effect across the post-thrombectomy fibrinolytic strategies represented in these trials, not as evidence that alteplase, tenecteplase, and urokinase are therapeutically interchangeable. Their differences in fibrin specificity, plasma half-life, and the dosing and administration regimens evaluated across trials may translate into agent-specific efficacy or safety profiles that cannot be resolved from the present trial-level evidence.

The hemorrhagic outcomes warrant a similar caution regarding what statistical homogeneity does and does not establish. Symptomatic and any intracranial hemorrhage both yielded point estimates above unity (RR 1.203 and 1.120, respectively), with confidence and prediction intervals compatible with no effect as well as clinically relevant excess risk. Earlier syntheses, including those of Palaiodimou and Doheim, likewise reported no statistically significant increase in these outcomes [15,16]. The present analysis extends that interpretation by explicitly incorporating the clinical heterogeneity of the underlying hemorrhagic definitions into the certainty assessment. Heidelberg-based criteria or classifications were used in five trials, ECASS III criteria in two, and trial-specific definitions in the remainder, with ascertainment windows also varying across studies. This heterogeneity was treated as a source of indirectness rather than statistical inconsistency within the GRADE framework and was a principal reason certainty of evidence for both hemorrhagic outcomes was rated low despite an observed I^2^ of 0.0% for each. A statistically homogeneous pooled estimate derived from non-uniform outcome definitions should therefore not be interpreted as evidence of clinical equivalence among those definitions.

The updated evidence can also be considered in light of a mechanistic distinction articulated more recently: between distal embolic territorial hypoperfusion, which arises from macroembolic fragments beyond the reach of the retrieval device and remains, in principle, susceptible to a locally infused fibrinolytic, and true ischemic-core no-reflow, a pericyte- and endothelium-mediated microvascular phenomenon unlikely to respond to any fibrinolytic agent regardless of route of administration [2,3].

This distinction provides a biologically coherent framework for interpreting the pattern observed across the randomized evidence in aggregate—a modest but statistically reproducible functional benefit in the absence of a clearly demonstrated overall excess in mortality or intracranial hemorrhage. Such a pattern is compatible with a treatment effect confined to a biologically responsive subset of patients rather than with uniform reversal of post-reperfusion microvascular injury across the treated population. A population-averaged functional benefit of this magnitude, with limited detectable impact on mortality or hemorrhagic risk, is what one would expect if adjunctive IAT acts principally by clearing distal macroembolic material in a subset of patients rather than by reversing established microvascular injury across the treated population as a whole. It should be emphasized that none of the nine included trials incorporated the perfusion-imaging phenotyping that would be required to test this hypothesis directly; it is offered here as a framework to guide future mechanistic and imaging-linked trials, not as a conclusion this trial-level synthesis can itself sustain.

Pharmacogenomic variation may represent an additional source of interindividual heterogeneity in fibrinolytic response. The PAI-1 5G/5G genotype has been associated with an increased risk of intracranial hemorrhage after intravenous fibrinolysis [27]; however, none of the randomized trials included in the present review reported genotype-level data. Whether genetic determinants of fibrinolysis influence the efficacy or safety of adjunctive IAT therefore remains unknown and warrants investigation in future pharmacogenomically informed trials.

Several methodological choices distinguish this update from its predecessors independent of the additional trials it incorporates. Every outcome was re-extracted from the final primary publication or official supplementary material rather than carried forward from earlier tabulations, and internal inconsistencies within primary reports—the ATTENTION-IA sICH percentages that do not correspond exactly to their stated denominators of 104 being one example—were documented rather than silently reconciled [5]. Sparse-event analyses used a treatment-arm continuity correction that accounts for unequal group sizes, thereby avoiding the potentially disproportionate influence of a constant correction on relative-effect estimates when treatment arms are unbalanced [23]. The cumulative meta-analytic framework provided a complementary perspective by characterizing the trajectory of the randomized evidence rather than its current pooled state alone, thereby distinguishing a mortality inference that remained broadly stable across the earlier evidence base from one that changed after incorporation of CHOICE-2. And the mRS 0–1 and mRS 0–2 thresholds were treated throughout as statistically dependent, nested outcomes rather than as independent endpoints open to informal cross-comparison, a distinction that clarifies rather than obscures the discordant pattern described above.

This review has several limitations that warrant consideration beyond the formal certainty-of-evidence assessments. The electronic bibliographic search included PubMed/MEDLINE, Embase, and CENTRAL, supplemented by backward and forward citation tracking and a separate search of ClinicalTrials.gov. In keeping with the source-verification approach used for quantitative extraction, unpublished outcome data were not sought directly from trial investigators when compatible numerical data were unavailable in the published record. This decision limited outcome-specific inclusion of IAT-TOP and CHOICE-2 and should be recognized as a limitation of the present synthesis. The analysis was necessarily conducted at the trial level rather than with individual participant data, precluding adjustment for baseline imbalances and the evaluation of within-trial treatment-effect modification. Analysis populations were not fully harmonized across studies: CHOICE and CHOICE-2 reported outcomes in treated-as-randomized populations after a small number of post-randomization exclusions, whereas most other trials used intention-to-treat or full-analysis-set populations; no attempt was made to impose common denominators through imputation. The heterogeneity of reported ordinal mRS estimands likewise precluded a defensible pooled ordinal analysis, with a consequent loss of information relative to approaches capable of exploiting the full disability distribution. Formal testing for small-study effects was not undertaken because no outcome-specific synthesis included ten or more studies; the absence of such testing should not be interpreted as evidence against publication or other missing-evidence bias. Finally, the randomized evidence remains geographically concentrated, with the large majority of participants enrolled in China or Spain; the extent to which these findings generalize to health systems with different stroke-care infrastructure, treatment pathways, and patient case-mix therefore remains uncertain.

## 5. Conclusions

This updated synthesis indicates that the efficacy and mortality components of the benefit–risk profile of adjunctive intra-arterial thrombolysis after successful or near-successful endovascular reperfusion have not evolved in parallel. The improvement in excellent functional recovery persisted as the randomized evidence base expanded and was supported by a pooled effect estimate of RR 1.266 (95% CI 1.135–1.412), a prediction interval excluding the null, and a meta-analytic Fragility Index of 15. In contrast, 90-day all-cause mortality remained statistically inconclusive in the primary synthesis. Its cumulative estimate changed after incorporation of CHOICE-2, and the boundary-optimized reverse-fragility analysis identified the earliest significance transition after 24 event-status modifications, in the direction of increased rather than reduced mortality with adjunctive IAT. This directional finding does not establish a treatment-related mortality excess; it indicates instead that the present absence of statistical significance should not be interpreted as affirmative evidence of mortality neutrality. The principal contribution of this update is therefore not another demonstration of an established efficacy signal, but a clearer characterization of the evidence accompanying that benefit. Cumulative meta-analysis shows how the functional and mortality estimates evolved as randomized evidence accrued, whereas fragility analysis provides a complementary measure of the stability and directionality of the corresponding statistical inferences. Taken together, these analyses support a reproducible and comparatively stable improvement in excellent functional recovery while preserving clinically important uncertainty regarding survival. Further clarification of that uncertainty will require additional randomized evidence with adequate information on mortality or individual-patient-data analyses capable of distinguishing a treatment-related effect from trial-specific differences in baseline risk and control-group outcomes. Until such evidence becomes available, the functional benefit of adjunctive IAT should be interpreted within a benefit–risk framework in which the remaining uncertainty surrounding mortality is neither overstated nor assumed away.

## Figures and Tables

**Table 1:** Characteristics of the Randomized Trials Included in the Systematic Review.

Trial	Setting and Design	Randomized Population	Vascular Territory and Reperfusion Criterion	Previous IVT and Intra-Arterial Regimen	Primary Efficacy Endpoint and Analysis Population
CHOICE (2022) [4]	Spain; 7 centers. Phase 2b, randomized, double-blind, placebo-controlled trial	121	ICA, M1/M2, or PCA; eTICI ≥ 2b50 *	IVT permitted. Alteplase 0.225 mg/kg; maximum 22.5 mg; IA infusion	mRS 0–1 at 90 days. Treated-as-randomized population: 61 vs. 52
ATTENTION-IA (2025) [5]	China; multicentre. Randomized, open-label, blinded-endpoint trial	208	Posterior circulation; VA, BA, or P1; eTICI 2b–3	IVT permitted. Tenecteplase 0.0625 mg/kg IA	mRS 0–1 at 90 days. Analysis population: 104 vs. 104
POST-TNK (2025) [6]	China; 34 hospitals. Randomized, open-label, blinded-outcome trial	540	Anterior circulation; eTICI 2c–3	Previous IVT not permitted. Tenecteplase 0.0625 mg/kg IA bolus	mRS 0–1 at 90 days. Randomized: 269 vs. 271; 90-day efficacy analysis: 269 vs. 270
POST-UK (2025) [7]	China; 35 hospitals. Randomized, open-label, blinded-endpoint trial	535	Anterior circulation; eTICI 2c–3	Previous IVT not permitted. Urokinase 100,000 IU IA, single dose	mRS 0–1 at 90 days. Assigned: 267 vs. 267; primary efficacy analysis: 266 vs. 266
ANGEL-TNK (2025) [8]	China; multicentre. Phase 3, randomized, open-label, blinded-endpoint trial	256	Anterior circulation; eTICI ≥ 2b	Previous IVT not permitted. Tenecteplase 0.125 mg/kg IA; slow infusion	mRS 0–1 at 90 days. Main analysis population: 126 vs. 129; one participant withdrew consent immediately after randomization
DATE (2025) [9]	China; 30 sites. Phase 1b/2a study; only the randomized phase 2a component was included	157 †	Anterior circulation; eTICI 2b–3 after EVT	Previous IVT not permitted. Tenecteplase 0.0313 or 0.0625 mg/kg IA	Dose and safety endpoints in randomized phase 2a. Intervention: 46 + 46; shared control: 65
PEARL (2025) [10]	China; multicentre. Randomized, open-label, blinded-endpoint trial	324	Anterior circulation; eTICI ≥ 2b50	IVT permitted. Alteplase 0.225 mg/kg; 15 min IA infusion	mRS 0–1 at 90 days. Randomized: 164 vs. 160; primary mRS analysis: 163 vs. 159
IAT-TOP (2026) [11]	China; 37 comprehensive stroke centers. PROBE randomized trial	247	Posterior circulation; acute BAO; eTICI ≥ 2b50	IVT permitted. Alteplase 0.225 mg/kg; maximum 22.5 mg; 15 min IA infusion	mRS 0–2 at 90 days. Full analysis set: 124 vs. 122
CHOICE-2 (2026) [12]	Spain; 14 stroke centers. Randomized, open-label, blinded-outcome trial	440	LVO after successful EVT; eTICI 2b50–3	IVT permitted. Alteplase 0.225 mg/kg; maximum 20 mg; 15 min IA infusion	mRS 0–1 at 90 days. Treated-as-randomized population: 214 vs. 219 (433 total)

* CHOICE: The reperfusion criterion was amended during the trial to eTICI ≥ 2b50. Eligible occlusion sites included the ICA, M1/M2 segments, and PCA; the trial was therefore considered a mixed, anterior-dominant population rather than an exclusively anterior-circulation cohort. † DATE: Only the randomized phase 2a component was included in the evidence synthesis. The two tenecteplase groups (0.0313 and 0.0625 mg/kg; n = 46 each) shared a control group of 65 participants; the preceding nonrandomized phase 1b dose-escalation component was excluded. Abbreviations: BA, basilar artery; BAO, basilar artery occlusion; eTICI, expanded Treatment in Cerebral Ischemia; EVT, endovascular thrombectomy; IA, intra-arterial; ICA, internal carotid artery; IVT, intravenous thrombolysis; LVO, large-vessel occlusion; M1/M2, first and second segments of the middle cerebral artery; mRS, modified Rankin Scale; P1, first segment of the posterior cerebral artery; PCA, posterior cerebral artery; PROBE, prospective randomized open-label blinded-endpoint; VA, vertebral artery.

**Table 2:** Summary of Meta-Analytic Findings and Certainty of Evidence.

Outcome	Evidence Base	Random-Effects RR (95% CI)	I^2^ (%)	95% Prediction Interval	GRADE Certainty
Excellent functional recovery (mRS 0–1 at 90 days)	8 RCTs	1.266 (1.135–1.412)	0.0	1.047–1.530	Moderate
90-day all-cause mortality	8 RCTs	1.036 (0.817–1.314)	22.9	0.815–1.318	Moderate
Functional independence (mRS 0–2 at 90 days)	8 RCTs	1.032 (0.981–1.086)	0.0	0.939–1.135	Moderate
Symptomatic intracranial hemorrhage (sICH)	8 RCTs	1.203 (0.806–1.796)	0.0	0.744–1.945	Low
Any intracranial hemorrhage (ICH)	6 RCTs	1.120 (0.938–1.337)	0.0	0.860–1.458	Low

Abbreviations: CI, confidence interval; GRADE, Grading of Recommendations Assessment, Development and Evaluation; ICH, intracranial hemorrhage; mRS, modified Rankin Scale; RCT, randomized controlled trial; RR, risk ratio; sICH, symptomatic intracranial hemorrhage. Note: Effect estimates are risk ratios from random-effects models with between-study variance estimated by restricted maximum likelihood and 95% CIs calculated using the Hartung–Knapp method. Prediction intervals were calculated according to the method specified for the updated analysis for syntheses including at least five studies. Certainty of evidence was assessed separately for each outcome using GRADE.

## Data Availability

All data analyzed in this study were obtained from previously published randomized controlled trials and their associated Supplementary Materials, as cited in the manuscript. No new primary data were generated in this study. The data supporting the findings of this meta-analysis are available in the cited publications and the Supplementary Materials of this article. Additional information related to the study is available from the corresponding author upon request.

## Supplementary Materials

The following supporting information can be downloaded at https://www.mdpi.com/article/10.3390/jcm15207777/s1, Figure S1: Any intracranial hemorrhage; Table S1: Outcome-Specific Trial Availability; Table S2: Companion Reports Linked to Included Randomized Trials; Table S3: Randomized Studies Without Analyzable Published Results at Search Closure; Table S4: Definitions and Ascertainment of Hemorrhagic Outcomes; Table S5: Trial-Specific Ordinal mRS Estimands and Disposition; Table S6: Trial-Exclusion Sensitivity Analyses; Table S7: Detailed RoB 2 Assessments of the Included Randomized Trials; Table S8: GRADE Certainty-of-Evidence Profile; Table S9: Cumulative Meta-Analysis of Excellent Functional Recovery and 90-Day All-Cause Mortality; Table S10: Meta-Analytic Fragility and Reverse-Fragility Analyses; Supplementary File S2: Completed PRISMA 2020 Checklist, including the PRISMA 2020 for Abstracts checklist.

## Abbreviations

The following abbreviations are used in this manuscript:

BA	Basilar Artery
BAO	Basilar Artery Occlusion
CI	Confidence Interval
ECASS	European Cooperative Acute Stroke Study
eTICI	expanded Treatment in Cerebral Ischemia
EVT	Endovascular Thrombectomy
FI	Fragility Index
GRADE	Grading of Recommendations Assessment, Development and Evaluation
IA	Intra-Arterial
IAT	Intra-Arterial Thrombolysis
ICA	Internal Carotid Artery
ICH	Intracranial Hemorrhage
IVT	Intravenous Thrombolysis
LVO	Large-Vessel Occlusion
M1/M2	First and Second Segments of the Middle Cerebral Artery
mRS	Modified Rankin Scale
OR	Odds Ratio
PAI-1	Plasminogen Activator Inhibitor-1
P1	First Segment of the Posterior Cerebral Artery
PCA	Posterior Cerebral Artery
PI	Prediction Interval
PRISMA	Preferred Reporting Items for Systematic Reviews and Meta-Analyses
PROBE	Prospective Randomized Open-Label Blinded-Endpoint
PROSPERO	International Prospective Register of Systematic Reviews
RCT	Randomized Controlled Trial
REML	Restricted Maximum Likelihood
RoB 2	Revised Cochrane Risk of Bias Tool for Randomized Trials
RR	Risk Ratio
sICH	Symptomatic Intracranial Hemorrhage
TNK	Tenecteplase
tPA	Tissue-Type Plasminogen Activator
TSA	Trial Sequential Analysis
VA	Vertebral Artery
