# Supplementary material for: Functional Benefit and Safety of Adjunctive Intra-Arterial Thrombolysis After Successful Endovascular Thrombectomy: An Updated and Cumulative Meta-Analysis of Randomized Trials
Source: J Clin Med. 2026 Oct 9;15(20):7777. doi: 10.3390/jcm15207777 (PMC13705011; doi:10.3390/jcm15207777)
Supplement: Supplementary file 1 [file jcm-15-07777-s001.zip › Supplementary Materials.pdf]

Supplementary Materials

Functional Benefit and Safety of Adjunctive Intra-Arterial Thrombolysis After Successful Endovascular Thrombectomy: An Updated and Cumulative Meta-Analysis of Randomized Trials

Supplementary Search Strategy and Study-Linkage Rules

The bibliographic search comprised PubMed/MEDLINE, Embase, and the Cochrane Central Register of Controlled Trials (CENTRAL), with 15 August 2026 retained as the evidence cutoff for the review. The PubMed/MEDLINE export comprised 652 records corresponding to 652 unique PMIDs. Searches were outcome-agnostic and used database-appropriate controlled vocabulary, where applicable, together with free-text terms relating to acute ischemic stroke, endovascular thrombectomy or endovascular treatment, intra-arterial/intraarterial thrombolysis or fibrinolysis, and the fibrinolytic agents alteplase, tenecteplase, and urokinase.

PubMed query: (("intra-arterial"[Title/Abstract] OR intraarterial [Title/Abstract]) AND (thrombolysis [Title/Abstract] OR fibrinolysis [Title/Abstract] OR alteplase [Title/Abstract] OR tenecteplase [Title/Abstract] OR urokinase [Title/Abstract]) AND (thrombectomy [Title/Abstract] OR "endovascular treatment"[Title/Abstract] OR "endovascular therapy"[Title/Abstract]) AND (stroke [Title/Abstract] OR "cerebral infarction"[Title/Abstract]))

CENTRAL search strategy: The CENTRAL search combined the following free-text search with exploded MeSH descriptors for *Ischemic Stroke*, *Thrombectomy*, and *Thrombolytic Therapy*. The final search was the union of the free-text set and the intersection of the three MeSH sets.

Free-text search: (stroke OR "acute ischemic stroke" OR "acute ischaemic stroke" OR "cerebral infarction" OR "large vessel occlusion" OR LVO) AND ("endovascular thrombectomy" OR thrombectomy OR "endovascular treatment" OR "endovascular therapy" OR EVT OR "endovascular reperfusion" OR "endovascular recanalization" OR "endovascular recanalisation") AND ("intra-arterial thrombolysis" OR "intraarterial thrombolysis" OR "intra-arterial fibrinolysis" OR "intraarterial fibrinolysis" OR "intra-arterial alteplase" OR "intra-arterial tenecteplase" OR "intra-arterial urokinase" OR alteplase OR tenecteplase OR urokinase)

MeSH search: [Ischemic Stroke] exploded AND [Thrombectomy] exploded AND [Thrombolytic Therapy] exploded.

Final CENTRAL search: free-text search OR MeSH search.

Embase search strategy:

('stroke'/exp OR stroke OR 'ischemic stroke'/exp OR 'ischemic stroke' OR 'ischaemic stroke'/exp OR 'ischaemic stroke' OR 'acute ischemic stroke'/exp OR 'acute ischemic stroke' OR 'acute ischaemic stroke'/exp OR 'acute ischaemic stroke' OR 'cerebral infarction'/exp OR 'cerebral infarction' OR 'large vessel occlusion'/exp OR 'large vessel occlusion' OR 'large-vessel occlusion' OR lvo) AND ('thrombectomy'/exp OR thrombectomy OR 'mechanical thrombectomy'/exp OR 'mechanical thrombectomy' OR 'endovascular thrombectomy'/exp OR 'endovascular thrombectomy' OR 'endovascular treatment'/exp OR 'endovascular treatment' OR 'endovascular therapy'/exp OR 'endovascular therapy' OR evt OR 'endovascular reperfusion'/exp OR 'endovascular reperfusion' OR 'endovascular recanalization'/exp OR 'endovascular recanalization' OR 'endovascular recanalisation') AND ('intra-arterial thrombolysis' OR 'intraarterial thrombolysis'/exp OR 'intraarterial thrombolysis' OR 'intra-arterial fibrinolysis' OR 'intraarterial fibrinolysis' OR 'intra-arterial alteplase' OR 'intraarterial alteplase' OR 'intra-arterial tenecteplase' OR 'intraarterial tenecteplase' OR 'intra-arterial urokinase' OR 'intraarterial urokinase' OR 'alteplase'/exp OR alteplase OR 'tenecteplase'/exp OR tenecteplase OR 'urokinase'/exp OR urokinase).

CENTRAL yielded 600 records, and the cutoff-eligible Embase bibliographic search yielded 5,797 records. The additional database searches identified no further published randomized trial meeting the prespecified eligibility criteria for quantitative synthesis. Accordingly, the randomized evidence base contributing to the meta-analysis remained unchanged.

ClinicalTrials.gov was searched separately through 15 August 2026 using a registry search combining "ischemic stroke" with (“intra-arterial” OR intraarterial), (thrombolysis OR fibrinolysis OR alteplase OR tenecteplase OR urokinase), and (thrombectomy OR “endovascular treatment” OR reperfusion OR recanalization OR recanalisation). Trial-registry records retrieved through the CENTRAL and Embase searches were also assessed against the same clinical eligibility criteria. Registry records were evaluated at the study level, requiring randomized allocation and adjunctive intra-arterial fibrinolytic therapy after the trial-defined qualifying angiographic reperfusion threshold in the endovascular reperfusion setting. After parent-study linkage and deduplication across registry and bibliographic sources, eight unique randomized parent studies without analyzable published results at the 15 August 2026 evidence cutoff were retained in the systematic-review record. Eligibility was determined from the prespecified clinical PICO rather than from publication-type tags or registry labels. Reports and registrations arising from the same randomized population were linked to a single parent study; protocols, corrections, secondary analyses, and duplicate registrations were therefore retained for study-level verification where relevant but were not counted as additional randomized trials.

Study-Selection Documentation

Study selection was documented at the record, report, and study levels in accordance with PRISMA 2020. Multiple publications arising from the same randomized population were linked to their corresponding parent trial and were not counted as independent studies. Protocol reports, secondary analyses, and corrections associated with included trials were retained where relevant for study identification, source verification, and risk-of-bias assessment. The resulting selection process is summarized in the PRISMA flow diagram (Figure 1); outcome-specific trial availability, companion publications, **and randomized parent studies without analyzable published results at search closure** are detailed in **Tables S1, S2, and S3,** respectively.

Supplementary Figures

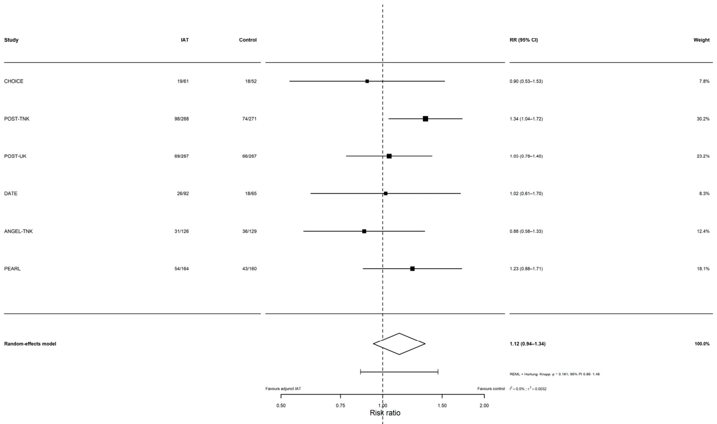

Figure S1. Any intracranial hemorrhage. Forest plot of the exploratory any-ICH analysis comparing adjunctive intra-arterial thrombolysis with control. Black squares represent individual trial risk-ratio estimates, with horizontal lines indicating their 95% confidence intervals. The diamond represents the pooled random-effects estimate, with its width indicating the corresponding 95% confidence interval. The vertical dashed line indicates the null effect (RR = 1). The horizontal interval beneath the pooled estimate represents the 95% prediction interval.

Supplementary Tables

Table S1. Outcome-Specific Trial Availability

Trial	mRS 0–1	90-day mortality	mRS 0–2	sICH	Any ICH	Outcome-level note
CHOICE	Included	Included	Included	Included	Included	—

Trial	mRS 0–1	90-day mortality	mRS 0–2	sICH	Any ICH	Outcome-level note
ATTENTION-IA	Included	Included	Included	Included	Not included	Compatible raw total any-ICH data were not available for the final synthesis.
POST-TNK	Included	Included	Included	Included	Included	—
POST-UK	Included	Included	Included	Included	Included	—
ANGEL-TNK	Included	Included	Included	Included	Included	—
DATE	Included	Included	Included	Included	Included	Randomized phase 2a TNK groups combined; shared control counted once.
PEARL	Included	Included	Included	Included	Included	Outcome-specific denominators retained as reported.
IAT-TOP	Not included	Not included	Included	Not included	Not included	Raw event counts required for mRS 0–1, mortality, sICH, and any-ICH syntheses were unavailable in the report used for extraction.
CHOICE-2	Included	Included	Not included	Included	Not included	Compatible raw mRS 0–2 and total any-ICH counts were unavailable.

Table S2. Companion Reports Linked to Included Randomized Trials

Parent trial	PMID	Report type	Disposition
ANGEL-TNK	39357896	Protocol	Linked to parent randomized study; not counted as an independent trial.
ANGEL-TNK	41136212	Secondary analysis	Linked to parent randomized study; not counted as an independent trial.
ATTENTION-IA	38124230	Protocol	Linked to parent randomized study; not counted as an independent trial.
CHOICE	31852410	Protocol	Linked to parent randomized study; not counted as an independent trial.
CHOICE	36054449	Prespecified imaging substudy	Linked to parent randomized study; not counted as an independent trial.
CHOICE-2	41339082	Protocol	Linked to parent randomized study; not counted as an independent trial.
IAT-TOP	39754489	Protocol	Linked to parent randomized study; not counted as an independent trial.
PEARL	39500603	Protocol	Linked to parent randomized study; not counted as an independent trial.
PEARL	40000092	Protocol correction	Linked to parent randomized study; not counted as an independent trial.
PEARL	40132858	Protocol correction	Linked to parent randomized study; not counted as an independent trial.
PEARL	42265863	Post hoc analysis	Linked to parent randomized study; not counted as an independent trial.
POST-TNK	39345180	Protocol	Linked to parent randomized study; not

Parent trial	PMID	Report type	Disposition
			counted as an independent trial.
POST-TNK	42565455	Secondary analysis	Linked to parent randomized study; not counted as an independent trial.
POST-UK	41573192	Protocol	Linked to parent randomized study; not counted as an independent trial.

Table S3. Randomized Studies Without Analyzable Published Results at Search Closure

Study	Registry ID	Status / evidence at search closure	Disposition
INSIST-IT	NCT05657457	Terminated; no analyzable numerical results available	Potentially eligible randomized evidence; not included in quantitative synthesis.
IA-SUCCESS	NCT06768138	Recruiting; no randomized outcome results available	Ongoing randomized evidence; not included in quantitative synthesis.
NEWLINE	NCT07727395	Registered before the 15 August 2026 evidence cutoff; no analyzable published results available	Ongoing randomized study of adjunctive IA alteplase after qualifying endovascular reperfusion; not included in quantitative synthesis.
TNK-FLOW	NCT06781385	Randomized study registered before the evidence cutoff; no analyzable published results available	Ongoing randomized evaluation of adjunctive IA tenecteplase in the endovascular reperfusion setting; not included in quantitative synthesis.
ALLY II	NCT06949228	Recruiting; no analyzable published results available	Ongoing randomized evaluation of adjunctive IA tenecteplase after qualifying post-thrombectomy reperfusion; not included in quantitative synthesis.
Reperfusion Completion Via Local Arterial Infusion	NCT07471282	Randomized study registered before the evidence cutoff; no analyzable published results available	Ongoing randomized evaluation of adjunctive IA tenecteplase after post-thrombectomy eTICI 2b to <3 reperfusion; not included in quantitative synthesis.

POST-UK2	ChiCTR2500105159	Prospectively registered randomized study; no analyzable published results available	Randomized evaluation of adjunctive IA urokinase after qualifying post-EVT reperfusion; not included in quantitative synthesis.
A Randomized, Prospective, Multicenter, Double-Blind, Placebo-Controlled Study to Evaluate the Safety and Efficacy of Intraarterial Injection of Recombinant TNK Tissue Plasminogen Activator (TNK) Following Partial Reperfusion (eTICI 2b50/67) in Mechanical Thrombectomy for Acute Large Vessel Occlusion	ChiCTR2600117118	Prospectively registered on 20 January 2026; recruitment scheduled from 26 January 2026 to 31 December 2029; no analyzable published results available at the 15 August 2026 evidence cutoff	Randomized evaluation of adjunctive IA tenecteplase versus placebo after qualifying eTICI 2b50/67 reperfusion; not included in quantitative synthesis.

Table S4. Definitions and Ascertainment of Hemorrhagic Outcomes

Trial	sICH definition	Assessment window	Any-ICH construct used in final synthesis
**CHOICE**	NIHSS worsening ≥4 points with intracranial hemorrhage	24 h	Any cerebral hemorrhage
**ATTENTION-IA**	Modified Heidelberg definition; adjudicated	≤36 h	Not synthesized: exact compatible total any-ICH denominator unresolved
**POST-TNK**	Modified Heidelberg Bleeding Classification	48 h	Any/radiological ICH within 48 h
**POST-UK**	Modified Heidelberg Bleeding Classification	48 h after thrombectomy	Any/radiological ICH within 48 h
**ANGEL-TNK**	Heidelberg bleeding classification	48 h	Any ICH within 48 h
**DATE**	ECASS III	24 h	Any ICH within 24 h
**PEARL**	Heidelberg criteria; definite/probable sICH	36 h	Any ICH within 36 h
**IAT-TOP**	Safety endpoint prespecified; exact definition not extractable from the main report used for extraction	≤48 h	Not synthesized
**CHOICE-2**	ECASS III; neurological deterioration ≥4 NIHSS points with ICH as predominant cause	36 h ± 24 h	Asymptomatic ICH reported; not relabeled as total any ICH

Table S5. Trial-Specific Ordinal mRS Estimands and Disposition

Trial	Ordinal approach / estimand	Disposition in review
**CHOICE**	Published common OR and exact mRS categories	Not pooled with non-equivalent ordinal estimands.
**ATTENTION-IA**	Adjusted common OR for favorable shift	Not pooled across all trials because ordinal estimands were not uniform.
**POST-TNK**	Adjusted generalized OR	Not pooled with proportional-odds common ORs.
**POST-UK**	Adjusted generalized OR	Not pooled with proportional-odds common ORs.
**ANGEL-TNK**	Wilcoxon–Mann–Whitney framework	Not pooled with common-OR estimands.
**DATE**	Dose-arm-specific generalized ORs	No valid published combined-arm ordinal estimate.
**PEARL**	Ordinal OR; exact categories available	Not pooled because cross-trial estimands remained non-equivalent.
**IAT-TOP**	Adjusted proportional-odds ordinal OR	Not pooled across all trials because estimands were not uniform.
**CHOICE-2**	Ordinal analysis reported; no extractable common OR in the report used for extraction	Not pooled.

Table S6. Trial-Exclusion Sensitivity Analyses

Outcome	Excluded trial	k	RR	95% CI	I² (%)	95% prediction interval
**mRS 0–1**	CHOICE	7	1.254	1.112–1.413	1.9	1.023–1.537
**mRS 0–1**	DATE	7	1.274	1.125–1.444	9.9	1.015–1.600
**mRS 0–1**	CHOICE-2	7	1.246	1.092–1.422	0.0	0.993–1.565
**90-day mortality**	CHOICE	7	1.057	0.827–1.351	20.1	0.803–1.391
**90-day mortality**	DATE	7	1.061	0.808–1.394	29.1	0.755–1.492
**90-day mortality**	CHOICE-2	7	0.984	0.801–1.209	0.0	0.778–1.245

Table S7. Detailed RoB 2 Assessments of the Included Randomized Trials

Trial	D1: Randomization process	D2: Deviations from intended interventions	D3: Missing outcome data	D4: Measurement of the outcome	D5: Selection of the reported result	Overall
CHOICE	Low	Low	Some concerns	Low	Low	Some concerns
ATTENTION-IA	Low	Low	Low	Low	Low	Low
POST-TNK	Low	Low	Low	Low	Low	Low
POST-UK	Low	Low	Low	Low	Low	Low
ANGEL-TNK	Low	Low	Low	Low	Low	Low
DATE	Low	Low	Low	Low	Some concerns	Some concerns
PEARL	Low	Low	Low	Low	Low	Low
IAT-TOP	Low	Low	Some concerns	Low	Low	Some concerns
CHOICE-2	Low	Low	Some concerns	Low	Low	Some concerns

Table S8. GRADE Certainty-of-Evidence Profile

Outcome	Evidence base	Risk of bias	Inconsistency	Indirectness	Imprecision	Missing-evidence / publication-bias concern	Certainty
**Excellent functional recovery (mRS 0–1 at 90 days)**	8 RCTs	Serious	Not serious	Not serious	Not serious	Not serious; formal small-study-effect testing was not feasible, and IAT-TOP did	Moderate

						not provide extractable mRS 0–1 event counts.	
**90-day all-cause mortality**	8 RCTs	Not serious	Not serious	Not serious	Serious	Not serious; formal small-study-effect testing was not feasible, and IAT-TOP raw mortality event counts were unavailable.	Moderate
**Functional independence (mRS 0–2 at 90 days)**	8 RCTs	Serious	Not serious	Not serious	Not serious	Not serious; CHOICE-2 did not provide compatible raw mRS 0–2 event counts.	Moderate
**Symptomatic intracranial hemorrhage (sICH)**	8 RCTs	Not serious	Not serious	Serious	Serious	Not serious; formal small-study-effect testing was not feasible, and IAT-TOP raw sICH event counts were unavailable.	Low
**Any intracranial hemorrhage (ICH)**	6 RCTs	Not serious	Not serious	Serious	Serious	Not serious; compatible outcome data were unavailable for three eligible trials.	Low

Abbreviations: GRADE, Grading of Recommendations Assessment, Development and Evaluation; ICH, intracranial hemorrhage; mRS, modified Rankin Scale; RCT, randomized controlled trial; sICH, symptomatic intracranial hemorrhage.

Note: Certainty was assessed separately for each outcome across the GRADE domains of risk of bias, inconsistency, indirectness, imprecision, and publication or reporting bias. Variation in hemorrhagic outcome definitions and ascertainment windows was considered under indirectness; sparse event counts and confidence intervals compatible with clinically relevant benefit and harm were considered under imprecision. Formal funnel-plot asymmetry testing was not undertaken because fewer than 10 studies contributed to each synthesis. Risk-of-bias downgrading was outcome-specific and reflected the relevance of the identified RoB 2 concerns to the outcome being assessed. Functional outcomes were downgraded by one level because concerns related to post-randomization exclusions, missing functional-outcome data, and selective outcome reporting were considered sufficiently relevant to their pooled estimates. These concerns were not judged to warrant downgrading the mortality or hemorrhagic outcomes for risk of bias; mortality was instead downgraded for imprecision, while the hemorrhagic outcomes were downgraded for indirectness and imprecision.

Table S9. Cumulative Meta-Analysis of Excellent Functional Recovery and 90-Day All-Cause Mortality

Publication block	Earliest online publication date	mRS 0–1, *k*	Pooled RR (95% CI)	I^2 (%)	Mortality, *k*	Pooled RR (95% CI)
CHOICE	10 February 2022	1	1.461 (0.989–2.160)	—	1	0.533 (0.186–1.529)
+ POST-TNK and POST-UK	13 January 2025	3	1.149 (0.895–1.476)	0.0	3	0.911 (0.529–1.570)
+ ATTENTION-IA	14 January 2025	4	1.163 (0.988–1.370)	0.0	4	0.940 (0.694–1.273)
+ DATE and ANGEL-TNK	5 July 2025	6	1.196 (1.048–1.365)	0.0	6	0.935 (0.786–1.113)
+ PEARL	13 October 2025	7	1.246 (1.092–1.422)	0.0	7	0.984 (0.801–1.209)
+ IAT-TOP	24 April 2026	7	1.246 (1.092–1.422)	0.0	7	0.984 (0.801–1.209)
+ CHOICE-2	7 May 2026	8	1.266 (1.135–1.412)	0.0	8	1.036 (0.817–1.314)

Abbreviations: CI, confidence interval; I^2, proportion of observed between-study variability attributable to heterogeneity; mRS, modified Rankin Scale; REML, restricted maximum likelihood; RR, risk ratio.

Note: Trials were ordered according to their earliest online publication date. Trials first published on the same date entered the cumulative evidence base simultaneously as a publication-date block. The initial CHOICE row represents a single-study estimate; between-study heterogeneity was therefore not estimable, and its 95% CI was calculated using the conventional log-RR normal approximation. From the first multi-study step onward, pooled estimates were obtained using the REML random-effects model applied in the updated analysis with Hartung–Knapp confidence intervals. IAT-TOP did not contribute raw event counts to either the mRS 0–1 or 90-day mortality cumulative synthesis; consequently, the cumulative estimates remained unchanged at the IAT-TOP publication step

Table S10. Meta-Analytic Fragility and Reverse-Fragility Analyses

Parameter	Result
Excellent functional recovery (mRS 0–1 at 90 days)	
Baseline pooled RR (95% CI)	1.266 (1.135–1.412)
Baseline p value	0.001
Fragility measure	Fragility Index (FI)
Index	15
Significance transition	Significant → non-significant
RR (95% CI) at transition	1.219 (0.995–1.494)
p value at transition	0.054
90-day all-cause mortality	
Baseline pooled RR (95% CI)	1.036 (0.817–1.314)

Baseline p value	0.735
Fragility assessment	Boundary-optimized bidirectional reverse-fragility analysis
Earliest significance transition identified by the search	24 one-patient event-status modifications
Direction of transition	Increased mortality with adjunctive IAT
RR (95% CI) at transition	1.174 (1.002–1.377)
p value at transition	0.048
Transition toward reduced mortality through 24 modifications	Not identified

Note: For excellent functional recovery, the FI was evaluated using the iterative event-status modification procedure described by Atal et al. For 90-day all-cause mortality, reverse fragility was evaluated using the boundary-optimized bidirectional search described in the Methods. The earliest significance transition identified by the mortality search occurred after 24 one-patient event-status modifications and was in the direction of increased mortality with adjunctive IAT. No transition toward reduced mortality was identified through the same modification depth. Because the mortality analysis used a finite-width search rather than exhaustive enumeration of all possible event-status configurations, the 24-modification result represents the earliest significance transition identified by the search and should not be interpreted as proof of a global mathematical minimum. Fragility measures characterize the stability of statistical significance and should not be interpreted as measures of treatment-effect magnitude, clinical importance, causality, or certainty of evidence.

Abbreviations: CI, confidence interval; FI, Fragility Index; IAT, intra-arterial thrombolysis; mRS, modified Rankin Scale; RR, risk ratio.
